# TGFβ1 in Cancer-Associated Fibroblasts Is Associated With Progression and Radiosensitivity in Small-Cell Lung Cancer

**DOI:** 10.3389/fcell.2021.667645

**Published:** 2021-05-20

**Authors:** Jiaqi Zhang, Jing Qi, Hui Wei, Yuanyuan Lei, Hao Yu, Ningbo Liu, Lujun Zhao, Ping Wang

**Affiliations:** ^1^Department of Radiotherapy, Tianjin Medical University Cancer Institute and Hospital, Tianjin, China; ^2^National Clinical Research Center for Cancer, Tianjin, China; ^3^Key Laboratory of Cancer Prevention and Therapy, Tianjin, China; ^4^Tianjin’s Clinical Research Center for Cancer, Tianjin, China; ^5^Department of Biochemistry and Molecular Biology, Tianjin Medical University Cancer Institute and Hospital, Tianjin, China; ^6^State Key Laboratory of Medicinal Chemical Biology (Nankai University), Tianjin, China; ^7^National Cancer Center/National Clinical Research Center for Cancer/Cancer Hospital, Shenzhen Hospital, Chinese Academy of Medical Sciences and Peking Union Medical College, Shenzhen, China

**Keywords:** small-cell lung cancer, cancer-associated fibroblasts, TGFβ1, prognostic marker, radiotherapy sensitivity, immune microenvironment

## Abstract

**Objective:**

Small-cell lung cancer (SCLC) is aggressive, with early metastasis. Cytokines secreted by cancer-associated fibroblasts (CAFs) within various tumors influences these features, but the function in particular of TGFβ1 (transforming growth factor beta 1) is controversial and unknown in SCLC. This study explored the influence of TGFβ1 in CAFs on the development, immune microenvironment, and radiotherapy sensitivity of SCLC.

**Methods:**

SCLC specimens were collected from 90 patients who had received no treatment before surgery. Tumor and tumor stroma were subjected to multiplex immunohistochemistry to quantitate TGFβ1 and other immune factors in CAFs. Cell proliferation and flow cytometry apoptosis assays were used to investigate associations between TGFβ1 and proliferation and radiotherapy sensitivity. The immune factors in tumors were detected by immunohistochemistry *in vitro* and *in vivo* (mice).

**Results:**

TGFβ1 levels on CAFs lower or higher than the median were found, respectively, in 52.2 and 47.8% of patients; overall survival of patients with TGFβ1-high levels (53.9 mo) was significantly longer than that of the TGFβ1-low group (26.9 mo; *P* = 0.037). The univariate and multivariate analyses indicated that a TGFβ1-high level was an independent predictor of increased survival time. TGFβ1-high levels in CAFs were associated with inhibition of growth, proliferation, antitumor immunity, and enhanced radiotherapeutic sensitivity and tumor immunity of tumor. TGFβ1-low levels promoted tumor cell growth and radiotherapy sensitivity *in vivo* and *in vitro*.

**Conclusion:**

High levels of TGFβ1 in CAFs were associated with longer overall survival in patients with SCLC and enhanced radiotherapy sensitivity.

## Introduction

The microenvironment of the tumor significantly influences it progression and the patient’s clinical outcome. Cancer-associated fibroblasts (CAFs) are the most important component of the tumor microenvironment that affects these characteristics (Chen and Song, 2019; Chen et al., 2019; Liao et al., 2019). CAFs originate mainly from the local resident fibroblasts of the tumor (Arina et al., 2016), which are activated by tumor cell-derived soluble factors such as transforming growth factor beta (TGFβ), platelet-derived growth factor (PDGF), and fibroblast growth factor 2 (FGF2) (Tauriello et al., 2018). In the tumor microenvironment, CAFs interact with other cells through direct cell-to-cell contact and by secreting cytokines such as TGFβ1, epidermal growth factor (EGF), hepatocyte growth factor (HGF), and interleukin 6 (IL-6) (Herrera et al., 2013; Hawinkels et al., 2014). Therefore, the interaction between CAFs and tumor cells influence tumor development and metastasis, and CAFs are considered a potential therapeutic target (Hanley et al., 2018).

Multiple cytokines may be necessary to activate resident fibroblasts to a CAF-like phenotype. Among them, TGFβ1 has shown the ability to induce a varied set of responses in cancer progression and metastasis. TGFβ1 acts as a tumor suppressor, by inhibiting cell proliferation and promoting apoptosis of tumor cells (Zhang et al., 2017). On the other hand, TGFβ1 induced cancer progression and metastasis and inhibited antitumor immunity, thus promoting cancer (Seoane and Gomis, 2017). The mechanics of this duality has not been resolved.

TGFβ1 is a crucial factor in interactions between cancer cells and surrounding stromal fibroblasts. In colorectal cancer, upregulation of TGFβ1 gene expression led to poor prognosis in patients with cancer (Itatani et al., 2019). Yet, many studies have shown that low TGFβ1 expression in tumor stroma was associated with the promotion of cancer (Chen et al., 2019; Hao et al., 2019).

There are no reports in the literature concerning associations between TGFβ1 on the surface of fibroblasts and patient prognosis, or clinicopathological factors, in small-cell lung cancer (SCLC). Correlations between TGFβ1 levels on CAFs and the progression, metastasis, and antitumor immunity in SCLC have not been well defined.

The present study investigated whether the TGFβ1 level in CAFs of resected tumors of patients with SCLC may be an indicator of prognosis, and furthermore the effect of this level on the proliferation and radiotherapeutic sensitivity of tumor cells and tumor immune status.

## Materials and Methods

### Patients

A retrospective review initially considered 138 patients with SCLC, who had been treated from January 2008 to December 2017 at our institution. For inclusion, no patient underwent preoperative pathologic examination or received other treatment prior to surgery; each received a postoperative pathologically proven diagnosis of SCLC; clinical data were complete and pathological samples were available; and every patient completed follow-up. Finally, 90 cases were enrolled.

All the patients were examined before surgery with computed tomography (CT) of the chest and upper abdomen, and in selected cases, brain magnetic resonance imaging (MRI) and total body positron emission tomography (PET)-CT. All the patients were classified according to the Veterans Administration Lung Study Group staging standard of the 2017 National Comprehensive Cancer Network (NCCN) guidelines, and the seventh edition of the tumor-node-metastasis classification system of the American Joint Commission on Cancer and the Union for International Cancer Control. We randomly divided 90 patients into training cohort (70% of the sample) and validation group (30% of the sample).

### Treatment and Follow-Up

The patients were given operative treatment; including 79, 6, and 5 who underwent lobectomy, pulmonary wedge resection, and pneumonectomy, respectively. Sixty-nine patients received adjuvant chemotherapy with cisplatin/carboplatin and etoposide after radical surgery; the median number of cycles in this group was 4 (range, 1–11).

Thirty-one patients received local mediastinal radiotherapy after surgery. The median prescribed dose was 54 Gy (40–60 Gy), delivered in 20–30 fractions (median, 27). Thirteen patients received prophylactic cerebral irradiation.

Patients were followed up every 3 months during the first 2 years after radiotherapy, every 6 months during the following 2 years, and annually thereafter. Ultrasonography, enhanced CT, MRI, or PET-CT were used to evaluate treatment efficacy during the follow-up.

### Tissue Microarray

The hematoxylin and eosin (H&E)-stained slides prepared from the tumor specimens of each patient were observed under a high-power microscope. Areas where tumor cells were densely packed were identified, and some tumor stromal tissues were preserved. The selected target tissues, with a diameter of about 3–5 mm, were circled with a black marker under the microscope. The position marked on the H&E-stained slide was also marked on the corresponding paraffin block surface. The selected area on the paraffin block was sampled with a biopsy needle, and a 2-mm tissue core was transferred to a recipient block.

### Multiplex Immunohistochemistry

Multiplex immunohistochemistry was performed with immune indicators in the tissue microarray. Markers were selected and apportioned in 2 panels, panel-A and panel-B, and included TGFβ1 and pan cytokeratin in each panel. In addition, the markers in panel A were: CD8; alpha-smooth muscle actin (α-SMA); and the forkhead/winged-helix transcription factor FOXP3 (forkhead box protein 3). In panel-B, additional markers were: CD3; programmed death ligand-1 (PD-L1); and cytotoxic T-lymphocyte associated antigen 4 (CTLA4) (Supplementary Figures 1A–C). Among them, α-SMA was used to label CAFs and α-SMA is regarded as the most widely used biomarker for identifying CAFs (Tao et al., 2017; Hanley et al., 2018; Ren et al., 2018; Sung et al., 2019), detailed information is shown in Supplementary Table 1. Through analysis of the above markers, the immune status of patients was judged and the associations among the markers were evaluated. The sections were stained with multiplex fluorescence by using a PANO 7-plex IHC kit (catalog 0004100100, Panovue, Beijing, China) in accordance with the manufacturer’s instructions.

The stained slides were scanned using a Polaris System (PerkinElmer, Waltham, Massachusetts, US) with the same exposure times at 200× magnification. InForm image analysis software (Version 2.4, PerkinElmer, Waltham, MA, United States) was used for unmixing multispectral images. In each slide, 5 random areas without necrosis or damage were selected and scanned.

### Immunohistochemistry

Immunohistochemical staining was performed according to standard protocol. Paraffin sections were placed at 37°C overnight, de-paraffinized in xylene, and rehydrated through a graded ethanol series. Antigen retrieval was performed at 100°C for 20 min with citrate buffer or EDTA (ethylenediaminetetraacetic acid) antigen retrieval solution (pH 8), depending on the antibody. After protein block, primary antibodies were incubation at 4°C overnight for 90 min (TGFβ1, Abcam, ab92486; PDL1, Novus, NBP1-76769; CD8, CST, 98941; FoxP3, CST, 12653; CD11b, Abcam, ab133357; CD86, CST,19589; CD56, CST, 99746; CD206, Proteintech, 60143-1-lg; PARP1, Abcam, ab191217; cleaved-PARP1, Abcam, ab32064).

Sections were incubated with labeled polymer horseradish peroxidase rabbit/mouse second antibody for 20 min. DAB staining and hematoxylin staining were performed. Sections were dehydrated through a series of ascending alcohols to xylene and mounted. CD8, CD56, and CD86 were used to identify CD8^+^ T cells, NK (natural killer) cells, and M1-type macrophages, respectively, FoxP3, CD11b, and CD206 were used to identify Treg, MDSC, and M2-type macrophages. The immunohistochemical staining was evaluated by light microscopy.

Immunohistochemical results were scored independently by 2 experienced pathologists, with at least 2 experts in agreement. A traditional immunohistochemical H score for indicators was used to evaluate the dyeing results in each tissue specimen, by the sum of relative intensity (0: negative expression; 1+: weak; 2+: distinct; 3+: very strong) of specific staining multiplied by the percentage of the area with positively stained cells. The patients were apportioned into two groups based on the median value of the scoring results: with the low (high) groups less than (greater or equal to) the median.

### Cell Culture and Medium

Cells of the human SCLC cell line H446 were cultured in RPMI-1640 (Gibco) supplemented with 10% fetal bovine serum (FBS). Cells of the human fetal lung fibroblast cell line HF-1 were cultured in Ham’s F-12K (Procell) supplemented with 10% FBS. Cells of the mouse Lewis lung cancer cell line LLC were cultured in DMEM (Gibco) with 10% FBS. Cells of the mouse squamous cell lung cancer cell line KLN205 were cultured in (minimum essential medium (MEM; Gibco) with 10% FBS, 1% non-essential amino acids, and 1 mM sodium pyruvate (all materials from Gibco). Cells of the mouse fetal fibroblast cell line MEF (mouse embryonic fibroblast) were cultured in DMEM with 10% FBS, 1% non-essential amino acids, and 1 mM sodium pyruvate (all materials from Gibco). All cell lines were cultured in a 37°C incubator with humidified atmosphere and 5% CO_2_.

Fibroblast cells were seeded into 6-well plates and cancer cells were seeded into transwell membrane inserts containing 0.4 μm pores. After cell adherence, the transwell inserts containing cells were placed in contact with fibroblasts for 96 h, and the activated fibroblasts were used in immunofluorescence cell staining, CCK-8 assay, clonogenic survival assay, flow cytometry apoptosis assay and *in vivo* experiments. To prepare conditioned medium of cultured CAFs, the CAFs were first cultured in general medium that contained 10% FBS, 1% non-essential amino acids, and 1 mM sodium pyruvate. When the cells were in the logarithmic growth phase, the general medium was replaced with serum-free medium. After 48 h, the supernatant was collected as conditioned medium and the cell fragments were removed by centrifugation (Kim et al., 2018; Sampson et al., 2018). The conditioned medium from the normal fibroblasts (NFs), and CAFs with knocked down or overexpressed TGFβ1, were collected in the same way.

### *In vivo* Experiments

All procedures with animals were conducted in accordance with the guidelines of the Laboratory Animal Ethics Committee of Tianjin Medical University Cancer Institute and Hospital.

The 1 × 10^6^ LLC cells, 3 × 10^6^ CAFs with stable knockdown of TGFβ1 (shTGFβ1) or stable overexpression of TGFβ1, and the control group (empty plasmid) were mixed well and implanted subcutaneously into the right flank of female BLC57 4-week-old mice. Six mice were used in each group. Tumor volume was monitored every 2 days by measuring the length and width of the tumor with calipers, and calculated as length × width^2^ × 0.5. Tumors were excised and weighed when the tumor length exceeded 2.5 cm, and then embedded in paraffin.

### Immunofluorescence Cell Staining

Cells were grown on coverslips for 24 h and fixed in 4% paraformaldehyde at room temperature and permeabilized with 0.1% Triton X-100. Cells were incubated with 5% bovine serum albumin (BSA) solution at 37°C for 30 min to block nonspecific interactions. Cells were incubated serially with anti-αSMA and anti-FAPα primary antibodies (abcam, ab32575, ab53066), at 4°C overnight, and then with secondary antibodies (Beyotime, P0176-1) at room temperature in the dark for 1 h. Cells were co-stained with DAPI (4′,6-diamidino-2-phenylindole) to detect nuclei. Immunofluorescence images were captured using FV10-ASW viewer software (Olympus, Tokyo, Japan).

### Lentiviral Transfection

Plasmid containing the validated short-hairpin RNAs (shRNAs) or overexpressed RNA-targeted TGFβ1 were cloned into the vectors GV493 and GV358, respectively (Genechem, Shanghai, China). Vectors used in this study contained green fluorescent protein (GFP) sequence. The target sequences of TGFβ1 for constructing lentiviral shRNA were 5′-CGGCAGCTGTACATTGACTTT-3′ in mouse cells, and 5′-CAGCAACAATTCCTGGCGATA-3′ in human cells. The lentiviral expression constructs and packaging plasmids mix were co-transfected into 293T cells to generate the recombinant lentivirus.

LLC, MEF, and HF-1 cells were plated in 6-well plates and cultured overnight. The complete medium containing infection enhancer (Gene, REVG004) were added to the cells. The lentiviruses in medium were added for 10-h incubation at 37°C. After screening, the transfection efficiency was examined by western blot and ELISA (enzyme-linked immunosorbent assay).

### Western Blot Assay

Western blot was used to evaluate TGFβ1 protein levels in cells after lentivirus transfection. Antibody against TGFβ1, Caspase 3 and PARP1 were purchased from Abcam (Cambridge, United Kingdom). GAPDH (glyceraldehyde-3-phosphate dehydrogenase) was used as the normalized control. Cells were washed twice with phosphate-buffered saline and lysed in protein lysate at 4°C for 30 min. A BCA kit (Thermo Fisher Scientific, 23225) was used to detected protein concentration. The protein was mixed with loading buffer and separated by SDS-PAGE and transferred onto PVDF membrane. After blocking, the membranes were incubated with the primary antibodies overnight. The membranes were then incubated with second antibodies (Santa Cruz, United States) and exposed using a chemiluminometer.

### ELISA

The supernatants of transfected cells were collected after 48 h. The concentration of TGFβ1 in the supernatants was determined using an ELISA kit (DAKEWE, 1217102) following the manufacturer’s instruction. The optical density was detected by a microplate reader (Thermo Fisher Scientific, United States) at 450 nm, and the TGFβ1 concentration in culture media was calculated according to the formula of the standard curve.

### CCK-8 Assay

LLC, KLN205, and H446 suspensions (500 cells/well) were added to 96-well plates (200 mL/well) and, respectively, cultured in general medium; MEF or CAF conditioned medium; or the conditioned mediums of transfected CAFs (shRNA TGFβ1, overexpressed TGFβ1, and the control group). Each group was prepared with 5 parallel wells. At days 1, 2, 3, 4, 5, 6, and 7 after seeding, 20 μL of CCK-8 solution (MedChemExpress, HY-K0301) was added to each well. After a 1-h incubation at room temperature in the dark, the absorbance was measured with an enzyme calibrator at 450 and 630 nm, and the optical density values were measured.

The CCK-8 assay was used to determine the effect of CAFs and CAFs expressing different levels of TGFβ1 on the sensitivity of tumor cells to radiotherapy. Cancer cells (2000 cells/well) were added to 96-well plates in a volume of 200 mL/well and received radiotherapy of 0, 2, 4, 6, or 8 Gy. After radiotherapy, the cell culture medium was replaced, respectively, by general medium, the conditioned medium from MEFs or CAFs, or the conditioned mediums of the transfected CAFs (shRNA TGFβ1, overexpress TGFβ1 and their control group). Seventy-two hours after radiotherapy, the optical density values were measured as described above.

### Clonogenic Survival Assay

LLC, KLN205, and H446 cells were seeded in 6-well plates with 200, 500, 1,000, 1,500, or 2,000 cells per well for colony formation. After incubation for 24 h, cells were subjected to 0, 2, 4, 6, or 8 Gy of radiation, and the medium replaced with a conditioned medium. Colonies were stained with crystal violet and counted after incubation intervals of 7 days. Colonies of ≥ 50 cells were scored.

### Flow Cytometry Apoptosis Assay

LLC, KLN205, and H446 cells were seeded in 6-well plates and co-cultured with the conditioned mediums of the transfected CAFs. After radiotherapy of 0, 2, 4, 6, or 8 Gy, cells were collected for apoptosis assay. Staining with APC-conjugated Annexin V and propidium iodide was performed to determine the percentage of cells undergoing apoptosis, and in accordance with the manufacturer’s protocol (BD Biosciences, Bedford, MA, United States). Each sample was analyzed using a BD FACSCanto II flow cytometer (BD Biosciences) with annexin V on the horizontal axis and propidium iodide on the vertical axis.

### Nomogram Construction

For predicting the 1-, 3-, 5-year survival rate for SCLC patients, a nomogram was constructed. The nomogram included TGFβ1, PDL1 and stage on the basis of the Cox regression model. Calibration plots were constructed to assess the consistency between the predicted and observed probabilities. Nomogram was performed using the package “rms” of R software.

### Statistical Analysis

Statistical analyses were performed using the Statistical Package for Social Sciences (SPSS24.0) and R software (version 3.5.3). Survival curves were analyzed using the Kaplan-Meier method. The immune indicators which significant associated with OS (*P* ≤ 0.05) were estimated by univariate Cox regression analysis. The prognostic factors were selected by LASSO-COX regression analysis and Cox multivariate regression model was established. Nomogram was formulated by using the package of rms in R version 3.5.3. Decision curve analysis (DCA) and time-dependent receiver operating characteristic (ROC) curves were established to assess the clinical practicality of the nomograms using package “survivalROC” and “stdca.R” of R software.

Analysis of variance was calculated using Student’s *t*-test or one-way analysis of variance. The chi-squared test was applied to analyze the association between TGFβ1 level and the clinicopathological features of the patients. Correlation among different immune markers and immune checkpoints was calculated using Pearson’s correlation test.

The primary end-point of the clinical research in this study was overall survival (OS), which was considered the time from the initiation of treatment to death or last follow-up. The secondary end-point was progression-free survival (PFS), calculated as the time from treatment initiation to first disease progression or last follow-up. All tests were two-sided, and *P* ≤ 0.05 were considered statistically significant.

## Results

### Patients and Clinical Prognostic Factors

The characteristics for all the 90 patients are listed in Table 1. Among the 90 patients, the OS and PFS were 53.3 and 38.2 months, respectively. The effects of clinical characteristics on patient survival are listed in Table 2. The results showed that patients with I-II stage SCLC had longer OS and PFS than did patients with III stage. Gender, age, and smoking status had no significant effect on the prognosis of patients.

**TABLE 1:** Clinical features of the study population.

Characteristics	No. of patients	TGFβ1 expression in CAFs		*P*-value
		High	Low	
Age (years)				0.990
≥ 65	32	15(48.1%)	17(51.9%)	
<65	58	28(48.0%)	30(52.0%)	
Gender				0.839
Male	66	31(47.4%)	35(52.6%)	
Female	24	12(50.0%)	12(50.0%)	
Smoking history				0.127
Non-smoker	16	11(68.7%)	5(31.3%)	
Smoker	74	32(43.2%)	42(56.8%)	
Clinical stage				0.080
I	41	21(51.4%)	20(48.6%)	
II	16	3(21.4%)	13 (78.6%)	
III	33	19(57.1%)	14(42.9%)	
Lymphatic metastasis				0.665
Yes	39	20(51.5%)	19(48.5%)	
No	51	23(46.5%)	28(53.5%)	
Distant metastasis after therapy				0.438
Yes	36	19(52.8%)	17(47.1%)	
No	54	24(44.4%)	30(55.5%)	

*CAFs, cancer associated fibroblasts.*

**TABLE 2:** Univariate and multivariate ananlysis about impact of clinical features on OS and PFS of 90 patients with SCLC.

Variable	Median OS		Median PFS	
	Months	*P*-value	Months	*P-*value
Age (years)		0.420		0.866
≥65	46.06		–	
<65	53.32		46.69	
Gender		0.129		0.544
Male	42.91		46.69	
Female	–		38.21	
Smoking history		0.24		0.570
Yes	42.91		–	
No	53.32		–	
Stage		0.036		0.035
I–II	53.88		46.69	
III	38.08		23.56	
Stroma TGFβ1 expression		0.037		0.095
High	53.88		38.21	
Low	26.90		18.30	

Multivariate ananlysis about impact of clinical features on OS and PFS				

**Variable**	**OS**		**PFS**	
	
	**HR (95%)**	***P*-value**	**HR (95%)**	***P*-value**

Stage	2.148 (1.034–4.460)	0.040	1.898 (0.894–4.030)	0.095
Stroma TGFβ1 expression	0.466 (0.228–0.951)	0.036	0.989 (0.978–1.000)	0.060

*OS, overall survival; PFS, progression-free survival.*

### TGFβ1 Level in CAFs and Associations With Cancer Progression

**FIGURE 1:**
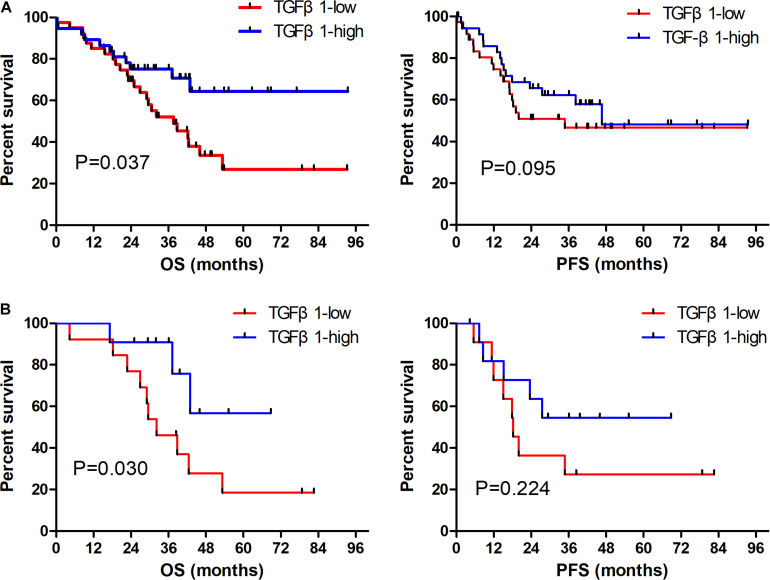
Prognostic role of TGFβ1 impact on OS and PFS in overall SCLC patients **(A)**; and in patients receiving radiation therapy **(B)**. OS, overall survival; PFS, progression-free survival.

The CAFs isolated from the patient tumor specimens were judged as TGFβ1-high or TGFβ1-low, i.e., higher or lower than the median for 34.3. The TGFβ1-high or TGFβ1-low groups comprised 43 (47.8%) and 47 (52.2%) patients, respectively. Patients in the TGFβ1-high group experienced a significantly longer OS and PFS compared with the TGFβ1-low group (Figure 1A). Specifically, the median OSs of the TGFβ1-high and -low groups were 53.9 (95% CI: 43.43–54.87) months and 26.9 (95% CI: 20.95–32.94) months, respectively (*P* = 0.037). The median PFSs of the TGFβ1-high and -low groups were 38.2 (95% CI: 19.09–42.29) months and 18.3 months (95% CI: 15.20–21.33; *P* = 0.095). The median OS of patients judged CD8-high (≥median: 26.9) in tumor stroma was 67.26 months, whereas that of CD8-low (< median: 26.9) patients was 43.5 months (*P* = 0.021). Patients in the PD-L1-high (≥ median: 1.4) group (in tumor) had a significantly longer median OS (58.1 mo) than did the PD-L1-low (< median: 1.4) patients (43.2 mo; *P* = 0.022). However, FoxP3 and CTLA-4 in tumor stroma appeared to have no prognostic effect within the study population. The univariate and multivariate Cox regression analysis showed that a TGFβ1-low level and PDL1- were independent prognostic factor for poor survival (Tables 3, 4).

**TABLE 3:** Univariate Cox regression analysis about the impact of immune indicators on OS and PFS of patients with SCLC.

Variable	OS			PFS		
	HR	95%CI	*P*-value	HR	95%CI	*P*-value
Stroma TGFβ1 expression	0.0.44	0.212–0.941	0.033	0.991	0.981–1.002	0.095
Tumor PDL1 expression	0.43	0.214–0.883	0.022	0.984	0.965–1.003	0.106
Stroma PDL1 expression	0.48	0.243–1.000	0.049	0.91	0.458–1.890	0.79
Stroma CD8 expression	0.993	0.981–1.006	0.093	0.994	0.988–1.000	0.054
Stroma FoxP3 expression	0.961	0.909–1.016	0.160	0.989	0.938–1.043	0.694
Stroma CTLA4 expression	0.972	0.770–1.227	0.814	0.890	0.677–1.171	0.406

*OS, overall survival; PFS, progression-free survival.*

**TABLE 4:** Multivariate LASSO-Cox regression analysis for OS in patients with SCLC.

Variable	OS		
	HR	95%CI	*P*-value
Stroma TGFβ1 expression	0.387	0.177–0.845	0.017
Tumor PDL1 expression	0.549	0.257–1.172	0.121

*OS, overall survival.*

The TGFβ1-high (TGFβ1-low) group included 24 (33) with stage I–II disease (55.8 cf. 70.2%), and 19 (14) with stage III disease (44.2 cf. 29.7%). In both the stage I–II and stage III groups, those stratified as TGFβ1-high had significantly longer OS compared with the TGFβ1-low (*P* = 0.030 and 0.032, respectively).

Thirty-one patients were treated with radiotherapy, specifically 15 (47.8%) in the TGFβ1-high group and 16 (52.2%) in the TGFβ1-low group. Patients treated with radiotherapy in the TGFβ1-high group experienced a significantly longer OS compared with those in the TGFβ1-low group (*P* = 0.030, Figure 1B).

To determine associations between TGFβ1 level in CAFs and other immune-related factors, levels of CD8, FoxP3, PD-L1, and CTLA4 in the tumor stroma and tumor were scored (Table 5). The TGFβ1 level (lower or higher than the median) in tumor stroma was positively associated with that of PD-L1 in tumor stroma and tumor (*P* ≤ 0.001, *R* = 0.428, 0.388), and CTLA4 in tumor stroma (*P* ≤ 0.001, *R* = 0.350), but not CD8 or FoxP3 in tumor stroma (*P* = 0.970; 0.249, respectively).

**TABLE 5:** Correlation of TGFβ1 expression in cancer associated fibroblasts with other immune indicators in 90 patients with SCLC.

Variable	*R*	*P*-value
FOXP3 expression in stroma	0.123	0.242
CD8 expression in stroma	−0.004	0.970
PDL1 expression in tumor cells	0.388	0.001
PDL1 expression in stroma	0.428	0.001
CTLA4 expression in stroma	0.350	0.001

### Risk Construction for OS and Univariate, Multivariate Analysis

The characteristics for patients in training and validation cohorts are listed in Supplementary Table 2. Based on the univariate analysis about the impact of immune indicators on OS, and the indicators with *P* ≤ 0.05 were used for model construction. The LASSO-COX regression model was used to generate the prognostic scoring system named Risk for OS in training cohort. We screened factors which significantly related with the prognosis of patients by LASSO-COX regression analysis and Risk was calculated through the formula: Risk is equal to the sum of the H score of the factors which significantly related with the prognosis multiplied by the corresponding regression coefficients (Supplementary Figure 2). The Risk included TGFβ1 in stroma and PDL1 in tumor. The patients were apportioned into two groups based on the median value of Risk: with the low (high) groups less than (greater or equal to) the median. Time-dependent ROC analyses were used to assess the prognostic accuracy of the Risk in training and validation cohort, the area under the curve (AUC) values at 1, 3, 5 year were 0.639, 0.783, 0.761 in training cohort and 0.627, 0.665, 0.772 in validation cohort, respectively. AUCs were used to assess the accuracy of prognosis. In training cohort, the prognosis of the low-Risk patients was significantly better than the high-Risk (*P* = 0.018). The same results were obtained in the validation cohort (*P* = 0.019) (Supplementary Figure 3). The univariate and multivariate Cox regression analysis showed Risk significantly related to the prognosis of patients in training and validation cohort (Supplementary Tables 3, 4). Nomogram was constructed for predicting the 1-, 3-, 5-year survival rate for SCLC patients (Supplementary Figure 4).

### Effect of CAFs on Tumor Growth and Radiation Sensitivity in Lung Cancer Cells

**FIGURE 2:**
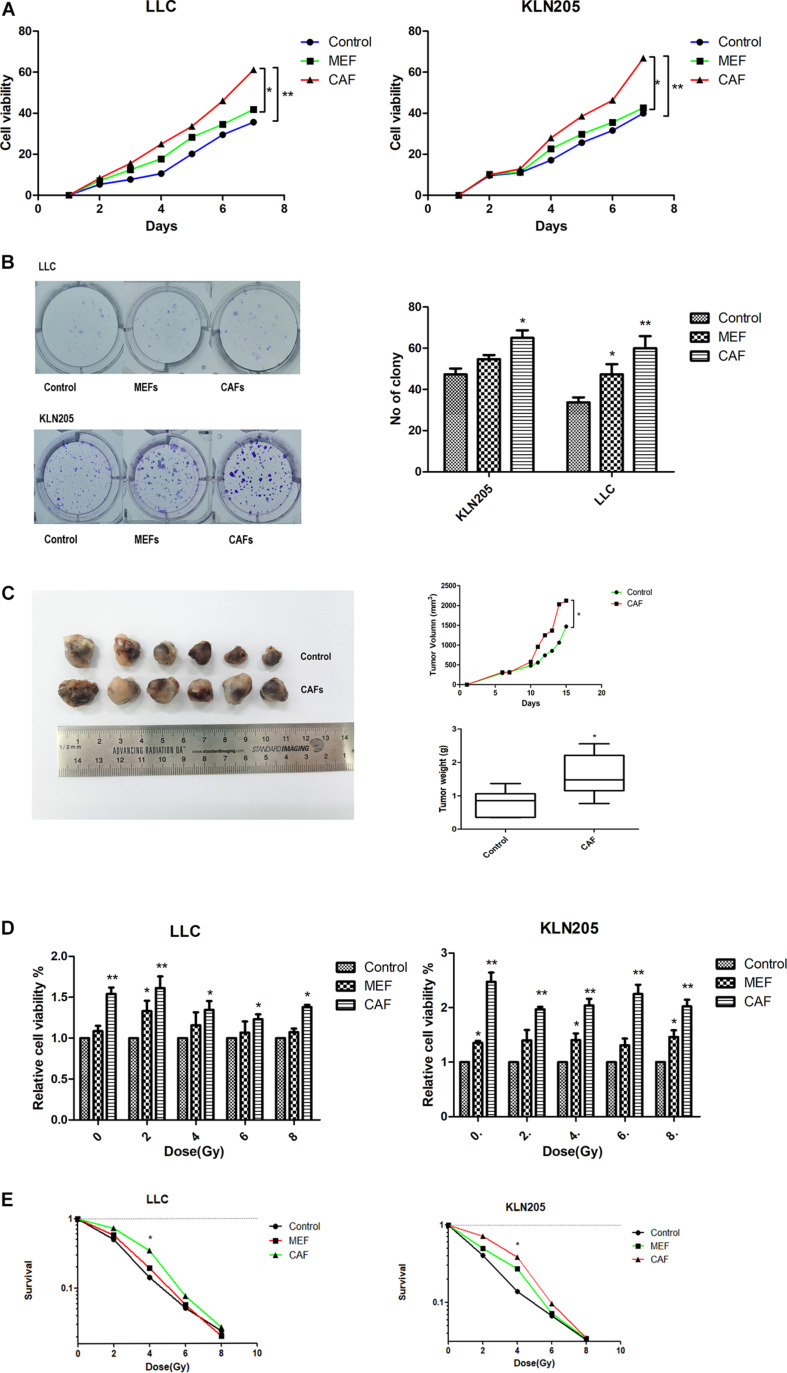
The impact of CAFs on lung cancer cell proliferation and radiosensitivity. **(A,B)** The impact of CAFs on cell viability and proliferation of lung cancer cells were examined by CCK-8 assay and clonogenic survival assay. **(C)** Tumor volume and weight of xenografts were measured with calipers and electronic balance. The mean tumor volume in CAF and Control groups were 2122.52 ± 365.95 mm^3^ and 1467.10 ± 436.41 mm^3^, respectively. The mean tumor weight in CAF and Control groups were 1.61 ± 0.84 g and 0.79 ± 0.44 g, respectively. **(D,E)** The impact of CAFs on cell viability and proliferation of lung cancer cells after radiotherapy for 0, 2, 4, 6, 8 Gy were examined by CCK-8 assay and clonogenic survival assay. Statistical significance is shown (*p < 0.05; **p < 0.01). MEF, mouse fetal fibroblast cell; CAF, cancer associated fibroblast; LLC, Lewis lung cancer cell; KLN205, mouse squamous cell lung cancer cell.

In the MEFs cultured with LLC and KLN205 conditioned medium, the levels of the markers α-SMA and FAP-α on the activated fibroblasts were higher than that of normal fibroblasts (Supplementary Figure 5). The proliferation of lung cancer cells was largely enhanced by coculture with CAFs or MEF cells, compared with lung cancer cells cultured alone. Cancer cells cultured with CAF-conditioned medium formed larger cell colonies than did cancer cells cultured alone (Figures 2A–C).

CAFs also had a certain effect on the radiotherapy sensitivity of tumor cells. After radiotherapy, the death of lung cancer cells cultured with CAF-conditioned medium was less than that cultured in general medium and MEF- conditioned medium, and the sensitivity to radiotherapy was lower (Figures 2D,E).

### Association Between Changes in TGFβ1 Level Produced by CAFs and Lung Cancer Cell Proliferation and Apoptosis

**FIGURE 3:**
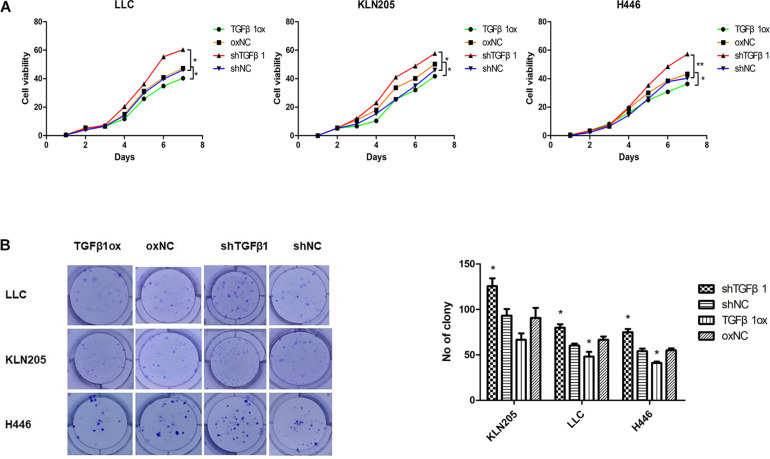
The impact of TGFβ1 level produced by CAFs on lung cancer cell proliferation. **(A,B)** TGFβ1-knocked down CAFs promoted lung cancer cells viability and proliferation. Cell viability and proliferation were examined by CCK-8 assay and clonogenic survival assay, TGFβ1- overexpression CAFs enhanced the viability and proliferation of lung cancer cells. Statistical significance is shown (^∗^*p* < 0.05; ^∗∗^*p* < 0.01). shNC and oxNC, control groups corresponding to knockdown and overexpression of TGFβ1 recombinant lentiviral infection; shTGFβ1 and TGFβ1ox, knockdown and overexpression of TGFβ1 recombinant lentiviral infection; LLC, Lewis lung cancer cell; KLN205, mouse squamous cell lung cancer cell; H446, human small cell lung cancer cell.

In the HF-1 and MEF cell lines, stable knockdown of TGFβ1 was accomplished by using TGFβ1-targeted shRNAs. Cells transduced with corresponding control scramble shRNA served as control cells. The efficiency of both knockdown and overexpression of TGFβ1 were confirmed by western blot and ELISA (Supplementary Figure 6). It was found that the proliferation and rates of colony formation of cancer cells cultured with TGFβ1-knocked down CAF-conditioned medium were significantly higher than that of the control cells, but there was a decrease in the growth of cancer cells cultured with over-expressed TGFβ1 CAF-conditioned medium (Figure 3). According to the western blot assay, TGFβ1 overexpression in CAFs led to a significant increase in the expression of PARP1 and cleaved-caspase3, compared with CAFs with TGFβ1 knocked down (Supplementary Figure 7). Meanwhile, The TGFβ1 level in tumor stroma was positively associated with both PARP1 and cleaved-PARP1 in tumor by immunohistochemical analyses (*P* ≤ 0.001, *R* = 0.430; *P* = 0.064, *R* = 0.206).

### Association Between Changes in TGFβ1 Expressed in CAFs and the Radiotherapy Sensitivity of Lung Cancer Cells

**FIGURE 4:**
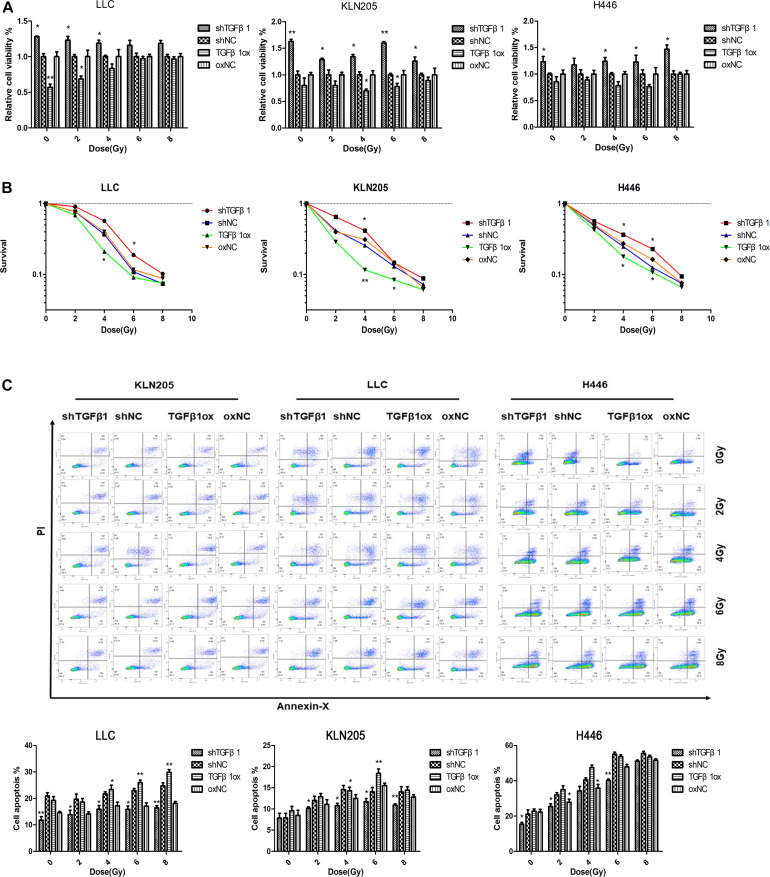
TGFβ1 level produced by CAFs affected the radiosensitivity of lung cancer cells. **(A,B)** CAFs with TGFβ1 knocked down inhibited the radiosensitivity of lung cancer cells after radiotherapy of 0, 2, 4, 6, or 8 Gy detected by CCK-8 assay and clonogenic survival assay, CAFs with TGFβ1 overexpression enhanced the radiosensitivity of lung cancer cells. **(C)** TGFβ1-knocked down CAF inhibited the apoptosis of tumor cells after radiotherapy and the apoptosis of tumor cells were induced after co-cultured with TGFβ1 overexpression CAFs. Cell apoptosis rate was examined by Annexin V/PI staining and flow cytometry assays. Statistical significance is shown (*p < 0.05; **p < 0.01). shNC and oxNC, control groups corresponding to knockdown and overexpression of TGFβ1 recombinant lentiviral infection; shTGFβ1 and TGFβ1ox, knockdown and overexpression of TGFβ1 recombinant lentiviral infection; LLC, Lewis lung cancer cell; KLN205, mouse squamous cell lung cancer cell; H446, human small cell lung cancer cell.

The clinical results showed that patients in the TGFβ1-low group had significantly shorter OS after radiotherapy than did the patients in the TGFβ1-high group. In the *in vitro* experiments, the CCK-8 cell proliferation and clonogenic survival assays suggested that CAFs with TGFβ1 overexpression led to higher cell activity and cell clone formation rate compared with CAFs with TGFβ1 knockdown after radiotherapy. According to the apoptosis assay, after radiotherapy, TGFβ1 overexpression in CAFs led to a significant increase in the proportion of apoptotic cells and the expression of PARP and cleaved-caspase3, compared with CAFs with TGFβ1 knocked down (Figure 4).

### Effect of TGFβ1 Level in CAFs on Tumor Growth of Lung Cancer Cells *in vivo*

In a mouse xenograft model, LLC cells injected together with CAFs led to greater growth of tumor relative to the group that received LLCs alone.

**FIGURE 5:**
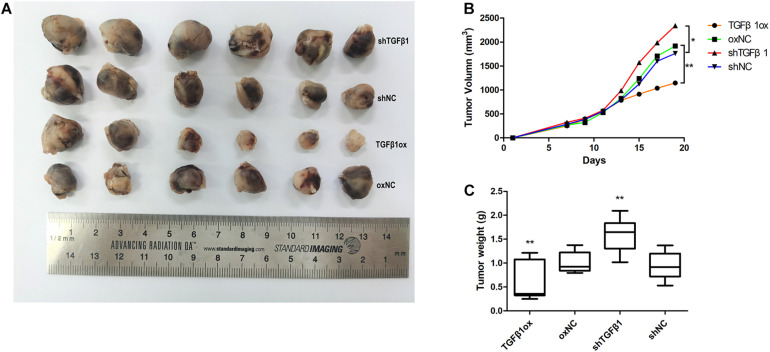
Tumor volume and weight of xenografts were measured with calipers and electronic balance. **(A)** TGFβ1 level in CAFs affected the proliferation of lung cancer cells xenografts. **(B)** The mean tumor volume in shTGFβ1, shNC, TGFβ1ox and oxNC were 2341.62 ± 305.32 mm^3^, 1760.65 ± 428.63 mm^3^, 1143.87 ± 166.57 mm^3^, and 1917.78 ± 415.77 mm^3^, respectively. **(C)** The mean tumor weight in shTGFβ1, shNC, TGFβ1ox and oxNC were 1.59 ± 0.57 g and 0.94 ± 0.41 g, 0.59 ± 0.23 g and 1.01 ± 0.22 g, respectively. Statistical significance is shown (*p < 0.05; **p < 0.01). shNC and oxNC: control groups corresponding to knockdown and overexpression of TGFβ1 recombinant lentiviral infection; shTGFβ1 and TGFβ1ox: knockdown and overexpression of TGFβ1 recombinant lentiviral infection.

The tumors in mice injected with LLC cells and CAFs with TGFβ1 knockdown grew significantly faster than that of LLC cells mixed with CAFs without TGFβ1 knockdown. Moreover, the tumors in mice injected with LLC cells with CAFs overexpressing TGFβ1 grew significantly slower compared with the controls. The tumors formed by LLC cells and TGFβ1-knockdown CAFs were approximately twice as large as those formed by LLC cells with CAFs overexpressing TGFβ1 (Figure 5).

LLC cells with TGFβ1 knocked down were mixed with CAFs with TGFβ1 knocked down, or CAFs with overexpressed TGFβ1, and the results were in accord with those reported above.

### Correlation Between TGFβ1 Levels in CAFs and Tumor Immune Microenvironment

**FIGURE 6:**
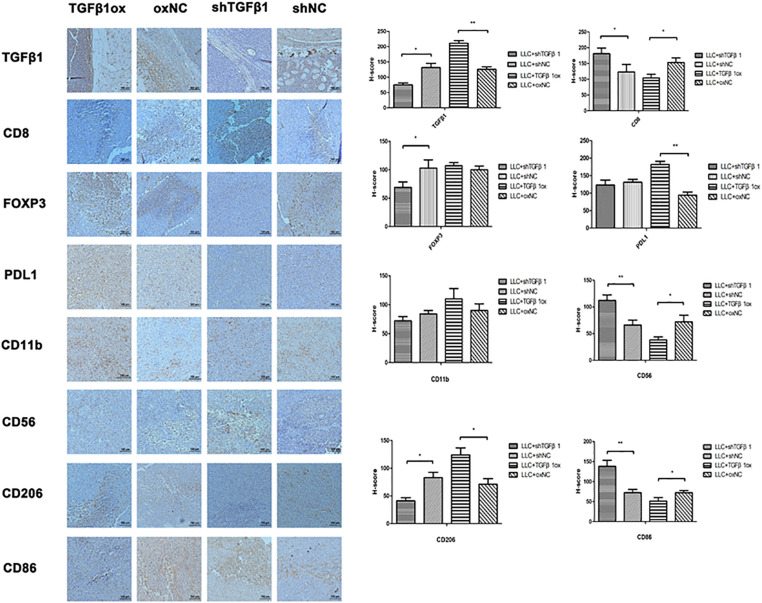
The impact of different TGFβ1 level in CAFs on tumor immunology. Immumohistochemical staining of lung cancer tissues in mice for TGFβ1, CD8, FOXP3, PDL1, CD56, CD11b, CD86, and CD206. TGFβ1 overexpression in CAFs inhibited the antitumor immunity of lung cancer xenograft. Statistical significance is shown (^∗^*p* < 0.05; ^∗∗^*p* < 0.01). shNC and oxNC, control groups corresponding to knockdown and overexpression of TGFβ1 recombinant lentiviral infection; shTGFβ1 and TGFβ1ox, knockdown and overexpression of TGFβ1 recombinant lentiviral infection.

The TGFβ1 level of CAFs in tumor tissues was positively associated with the levels of FoxP3, PD-L1, and CD206, but inversely associated with that of CD8, CD56, and CD86; no association was found with CD11b (Figure 6). Therefore, TGFβ1 in CAFs had an inhibitory effect on antitumor immunity, which was consistent with the SCLC specimens.

## Discussion

SCLC is characterized by rapid progression and early dissemination, and 80–90% of patients with SCLC receive a first diagnosis after the disease is advanced or extensive (Saltos et al., 2020). A diagnosis of SCLC still relies on small biopsies and cytological sample detection, and reports regarding the distribution and prognostic role of potential molecular biomarkers are limited (George et al., 2015).

In general, cancer cells and the cells of the tumor microenvironment interact, including the recruitment and activation of stromal cells by tumor cells. The microenvironment is crucial to tumor initiation, progression, metastasis, and drug resistance (Hinshaw and Shevde, 2019). In the present study, clinical samples obtained by surgical resection from patients with SCLC were analyzed for the multiplex immunohistochemistry of TGFβ1, immune-related proteins, and the immune checkpoint proteins, PD-L1 and CTLA4. The data suggest that TGFβ1 in CAFs are prognostic in SCLC and this result has been verified by LASSO-Cox regression model and nomogram. During tumor progression and concerning tumor immunity, the role of TGFβ1 is pivotal. The dual complexity of the functions of TGFβ1 in tumors, and the link between tumor progression and TGFβ1 in cancer stroma has warranted profound investigation (Zarzynska, 2014). The present study focused on TGFβ1 in CAFs and its growth-promoting activity in lung cancer cells *in vivo* and *in vitro*. It was found that TGFβ1 levels in CAFs that were lower than the median were sufficient to promote cancer cell proliferation and inhibit cell apoptosis, while higher TGFβ1 levels inhibited antitumor immunity. To our best knowledge, this study is the first to report that TGFβ1 in CAFs is an independent prognostic factor for patients with SCLC, especially in patients who are immune checkpoint–positive. This suggests that TGFβ1 signaling in fibroblasts is involved in the immune response in patients with SCLC.

The tumor microenvironment is a bidirectional, dynamic, and intricate network composed of tumor cells and surrounding stromal tissues. These include immune cells, fibroblasts, endothelial cells, extracellular matrix, cytokines, chemokines, and receptors (Hinshaw and Shevde, 2019; Vitale et al., 2019). Every factor in this network has an effect on tumor cells, and figures importantly in the tumor microenvironment (Piersma et al., 2020).

CAFs have also attracted more and more attention. Murata et al. (2011) found that CAFs have a key onco-supportive function in the development and progression of uterine cervical cancers. Paauwe et al. (2018) reported that CAFs contribute to colorectal cancer progression and metastasis, which is in agreement with other reports concerning breast and pancreatic cancers (Hwang et al., 2008; Ren et al., 2019; Wen et al., 2019). Yet, reports concerning CAFs in SCLC are rare. Using coculture and mouse model experiments, the current study revealed that the proliferation and tumorigenicity of SCLC cells was enhanced after co-culture with CAFs.

The complexity of the effects of TGFβ1 in tumor stroma on tumor cells and the mechanisms involved require further elucidation. In the current study, knockdown of TGFβ1 in CAFs was associated with greater proliferation and colony formation in co-cultured tumor cells. The TGFβ signaling pathway has been reported as a cancer suppressor (Yang et al., 2020). TGFβ1 suppressed tumor development by regulating the cell cycle, via increasing levels of cyclin-dependent kinase inhibitors (e.g., p15InK4b and p21Waf1/Cip1) and reducing c-Myc (Seoane and Gomis, 2017). Pan et al. (2006) reported that higher levels of TGFβ1 in tumor cells reduced the tumor growth of glioblastoma cells. These reports were confirmed by Liu et al. (2019).

The current study found that differences in TGFβ1 levels in CAFs appeared to affect the tumor immune microenvironment. High levels of TGFβ1 were positively associated with PD-L1, CD206, and FoxP3 levels in tumor tissues, but inversely with that of CD8, CD56, and CD86. In the clinical samples, the group with higher-than-the median TGFβ1 in CAFs had higher levels of PD-L1 and CTLA4, but no significant effect was shown by FOXP3 or CD8.

TGFβ has been reported to inhibit tumor immunity in many tumors (Batlle and Massagué, 2019). Castriconi et al. (2013) showed that neuroblastoma cells regulate the chemokine receptor repertoire of NK cells by releasing TGFβ1. FoxP3 levels correlated with TGFβ1 levels, and high TGFβ1 levels induced the transformation of T cells into Tregs, which is also present in skin cutaneous melanoma and breast cancer (Ravi et al., 2018). In addition, many studies have shown that TGFβ1 was associated with a reduction in pore forming protein (PFP) in CD8 cytotoxic T cells and weakened the killing activity of T cells (Batlle and Massagué, 2019). Similar results were found in the current study. However, the effect of TGFβ1 in CAFs on tumor immunity was inconsistent with the prognosis of clinical patients. This may be because the regulation of SCLC growth is dominated by the proliferation of tumor cells, a result shown in the present study. Combined immunotherapy is an alternative for patients with SCLC, due to the suppressive effect of TGFβ1 on antitumor immunity.

Radiotherapy is an important first-line treatment for SCLC (Nesbit et al., 2019; Wang et al., 2019). Sensitivity to radiation is an important factor that affects cure, through apoptosis and necrosis of tumor cells. In the present study, overexpression of TGFβ1 in CAFs appeared to enhance the apoptosis of lung cancer cells after radiotherapy. The mechanisms of TGF-β1 induced apoptosis has been reported in many studies include an increase in the expression of death-associated protein kinase (DAPK) (Jang et al., 2002) and TGF-β-inducible early response gene 1 (TIEG1) (Zhang et al., 2017). Additionally, after radiotherapy the proliferation of lung cancer cells that had been co-cultured with TGFβ1-knockdown CAFs was greater than that of the TGFβ1-overexpression CAFs. Among the patients who received radiotherapy, those in the TGFβ1-high group had a better prognosis than did those in the TGFβ1-low group. Therefore, the knockdown of TGFβ1 in CAFs may have enabled the SCLC cells to overcome apoptosis and promote the development of radiotherapy resistance. Few studies have suggested an association between the level of TGFβ1 in CAFs in tumors and the sensitivity to radiotherapy of tumors. Dickson et al. (2000) analyzed pretreatment plasma TGFβ1 levels and assessed the tumor control and late morbidity following radiation therapy in carcinoma of the cervix, found that an underlying weak relationship of TGFβ1 levels. The current results suggest a direct link between TGFβ1 and tumor radiosensitivity.

The role of TGFβ1 in cancer is complex and has been reported in many studies (Zarzynska, 2014; Seoane and Gomis, 2017). As tumor suppressor factors, TGFβRII or SMAD4 knockout significantly accelerated progression of malignant tumors; examples include APC inactivation in intestinal polyps, PyMT-induced breast cancer, and pancreatic injury induced by Kras (Biswas et al., 2004; Forrester et al., 2005; Ijichi et al., 2006; Munoz et al., 2006). On the other hand, TGFβRI activated in ErB2/HER2-positive patients with breast cancer slowed the development of tumor and reduced tumor volume (Muraoka et al., 2003; Siegel et al., 2003). This suggests that TGFβ1 signaling may control tumor growth. In the current study, we also found that the level of TGFβ1 in CAFs was associated with the prognosis of patients with SCLC, and tumor cell progression, multivariate analyses indicated that TGFβ1 was an independent indicator for prognosis in all cohorts. These incongruous reports suggest that the functions of TGFβ1 are complicated and may be specific for different tumor types. The effect TGFβ1 may differ by tumor types, either by inhibiting or promoting tumor growth. The mechanisms by which TGFβ1 affects SCLC cell development remain unclear, and further studies are required.

There are also some limitations in this study. This study was retrospective and enrolled samples in this study were derived from tumor tissues pathologically confirmed as SCLC after surgery. However, surgery is not an standard approach for SCLC, it is difficult to collect large numbers of histological samples. When surgical samples are not available, TGFβ1 can be measured with biopsy samples. Chemoradiotherapy has become the standard treatment for SCLC, therefore, further clinical trials need to be carried out to validate the results of this study in SCLC patients with chemoradiotherapy is required. Moreover, more complex mechanism of TGFβ1 expression in CAFs affecting the apoptosis of lung cancer cells requires further research.

## Conclusion

This study is the first to show that high TGFβ1 levels in CAFs may be an independent prognostic factor indicating good prognosis in patients with SCLC. In addition, the low TGFβ1 level in CAFs appeared to enhanced tumor cell development, radioresistance, and anti-tumor immunity, calling for different treatments. These results provide a basis for using TGFβ1 in CAFs as a novel predictor of prognosis in SCLC. Moreover, targeting TGFβ1 in CAFs may lead to a new therapeutic strategy to improve the survival outcomes of patients with SCLC.

## Data Availability Statement

The raw data supporting the conclusions of this article will be made available by the authors, without undue reservation.

## Ethics Statement

The studies involving human participants were reviewed and approved by Tianjin Medical University Cancer Institute and Hospital. The patients/participants provided their written informed consent to participate in this study. The animal study was reviewed and approved by Tianjin Medical University Cancer Institute and Hospital.

## Author Contributions

JQZ was the main authors of the manuscript. JQ, HW, NBL, HY, and PW made substantial contributions to the conception and design of the study. The sample collection was performed by JQ, YL, and LZ gave the final approval and revision of the manuscript. All authors read and approved the final manuscript.

## Conflict of Interest

The authors declare that the research was conducted in the absence of any commercial or financial relationships that could be construed as a potential conflict of interest.
