# Supplementary material for: TGFβ1 in Cancer-Associated Fibroblasts Is Associated With Progression and Radiosensitivity in Small-Cell Lung Cancer
Source: Front Cell Dev Biol. 2021 May 20;9:667645. doi: 10.3389/fcell.2021.667645 (PMC8172974; doi:10.3389/fcell.2021.667645)
Supplement: Supplementary file 1 [file Table_1.DOCX]

| **Supplementary Table S1.** Antibodies and Staining Conditions. | | | | | | |  |
| --- | --- | --- | --- | --- | --- | --- | --- |
| Markers | Targets | Antibody source | Product code | Species | Dilution | Location | |
| **PANEL-1** |  |  |  |  |  |  |  |
| CD8 | Cytotoxic T cell | Abcam | ab93278 | Rabbit monoclonal | 1:200 | Cell membrane, secreted |  |
| TGFβ1 | Cytokines | Abcam | ab92486 | Rabbit polyclonal | 400ⅹ | Secreted, extracellular space |  |
| FoxP3 | Treg | Biolegend | 320201 | Mouse monoclonal | 1: 50 | Cell nucleus |  |
| PanCK | Tumor cell | Abcam | ab215838 | Mouse monoclonal | 1:200 | Cell membrane |  |
| α-SMA | CAF | Abcam | ab32575 | Rabbit monoclonal | 1:200 | cytoplasm |  |
| **PANEL-2** |  |  |  |  |  |  |  |
| CD3 | T cell | Abcam | Ab135372 | Rabbit monoclonal | 1:150 | Cell membrane |  |
| TGFβ1 | Cytokines | Abcam | ab92486 | Rabbit polyclonal | 400ⅹ | Secreted, extracellular space |  |
| PanCK | Tumor cell | Abcam | ab215838 | Mouse monoclonal | 1:200 | Cell membrane |  |
| CTLA4 | Checkpoint | Abcam, | ab227709 | Rabbit monoclonal | 1:100 | Cell membrane, cytoplasm |  |
| PD-L1 | Checkpoint | DAKO | M3653 | Mouse monoclonal | 1:50 | Cell membrane, secreted |  |

**Supplementary Table S2.** Clinical features of patients in the training and validation cohorts.

| Variable | Training cohort (%) | Validation cohort (%) |
| --- | --- | --- |
| Gender |  |  |
| Make | 50 (79.4%) | 16 (59.3%) |
| Female | 13 (20.6%) | 11 (40.7%) |
| Age (years) |  |  |
| ≥65 | 22 (34.9%) | 10 (37.0%) |
| ＜65 | 41 (65.1%) | 17 (63.0%) |
| Smoking history |  |  |
| Nonsmoker | 10 (15.8%) | 6 (22.2%) |
| Smoker | 53 (84.2%) | 21 (77.8%) |
| Clinical stage |  |  |
| Ⅰ-Ⅱ | 39 (61.9%) | 18 (66.7%) |
| Ⅲ | 24 (38.1%) | 9 (33.3%) |
| Lymphatic metastasis |  |  |
| Yes | 29 (46.0%) | 10 (37.0%) |
| No | 34(54.0%) | 17 (63.0%) |
| Distant metastasis after therapy |  |  |
| Yes | 24 (38.1%) | 12 (44.4%) |
| No | 39 (61.9%) | 15 (55.6%) |

Supplementary Table S3. Univariate Cox regression analysis of Clinical features and Risk with OS.

| Variable | Training cohort | | Validation cohort | |
| --- | --- | --- | --- | --- |
|  | HR (95%) | P value | HR (95%) | P value |
| Age (years) (≥65 vs. ＜65) | 0.967 (0.452-2.065) | 0.930 | 2.944 (0.720-12.045) | 1.133 |
| Gender (female vs. male) | 0.494 (0.173-1.414) | 0.189 | 0.477 (0.058-3.904) | 0.490 |
| Smoking history (yes vs. no) | 2.759 (0.827-9.201) | 0.099 | 0.752 (0.087-6.480) | 0.796 |
| TNM Stage (Ⅰ-Ⅱ vs. Ⅲ) | 2.267 (1.093-4.703) | 0.028 | 1.177 (0.214-6.485) | 0.852 |
| Risk | 2.3446 (1.131-4.86) | 0.022 | 1.110 (1.017-1.210) | 0.019 |

Supplementary Table S4. Multivariate Cox regression analysis of Clinical features and Risk with OS.

| Variable | Training cohort | | Validation cohort | |
| --- | --- | --- | --- | --- |
|  | HR (95%) | P value | HR (95%) | P value |
| TNM Stage (Ⅰ-Ⅱ vs. Ⅲ) | 1.158 (0.763-1.757) | 0.491 | - | - |
| Risk | 1.020 (1.002-1.039) | 0.031 | 1.110 (1.017-1.210) | 0.019 |
