# Supplementary material for: TGFβ1 in Cancer-Associated Fibroblasts Is Associated With Progression and Radiosensitivity in Small-Cell Lung Cancer
Source: Front Cell Dev Biol. 2021 May 20;9:667645. doi: 10.3389/fcell.2021.667645 (PMC8172974; doi:10.3389/fcell.2021.667645)

**Supplementary Figure**

**Supplementary Fig. S1A.**  Representative multiplex fluorescent immunohistochemistry images of all 8 markers (divided into 2 PANELs). Bar, 100 µm.


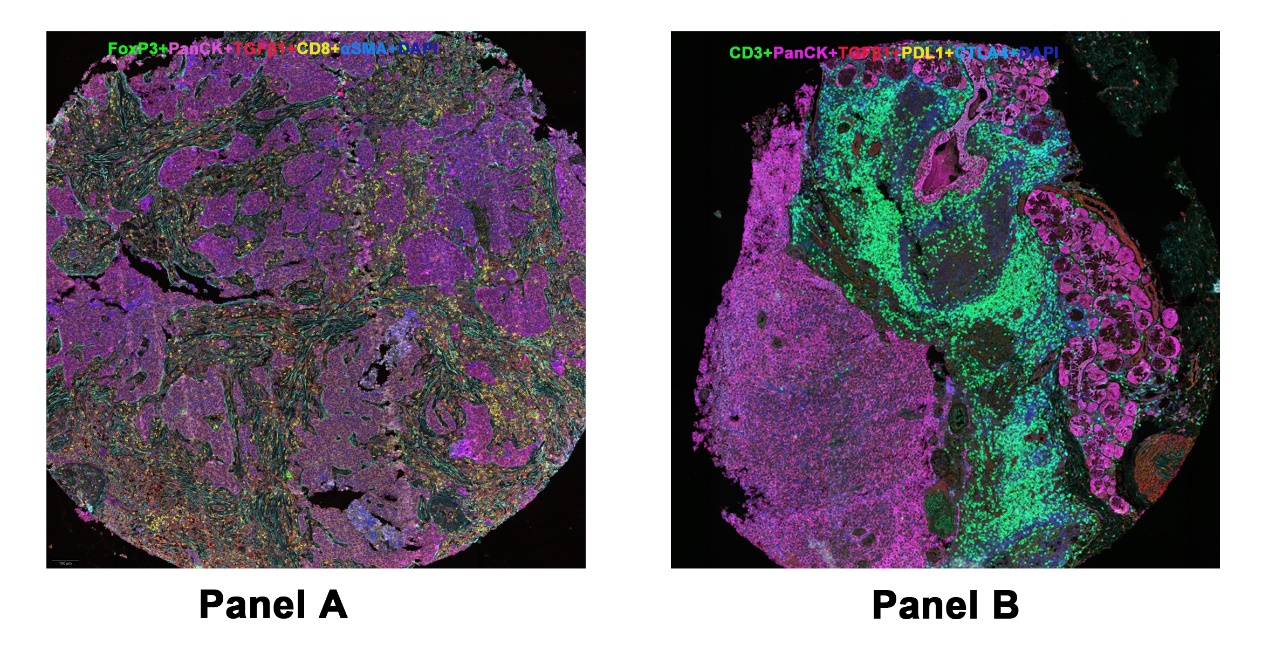


**Supplementary Fig. S1B.**  PANEL 1. Representative immunohistochemistry images of PANEL 1. Bar, 100 µm.


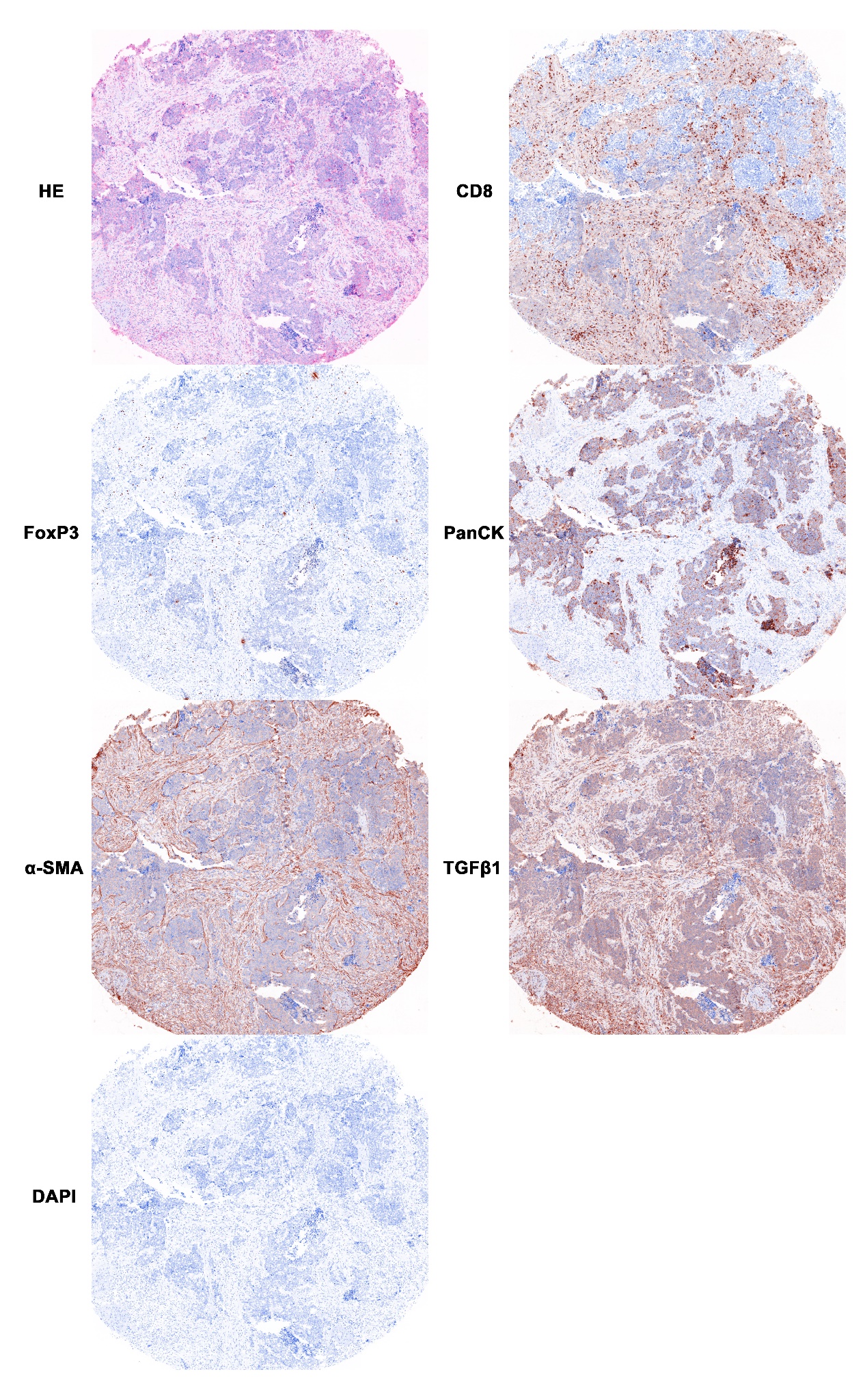


**Supplementary Fig. S1C.**  PANEL 2. Representative immunohistochemistry images of PANEL 2. Bar, 100 µm.


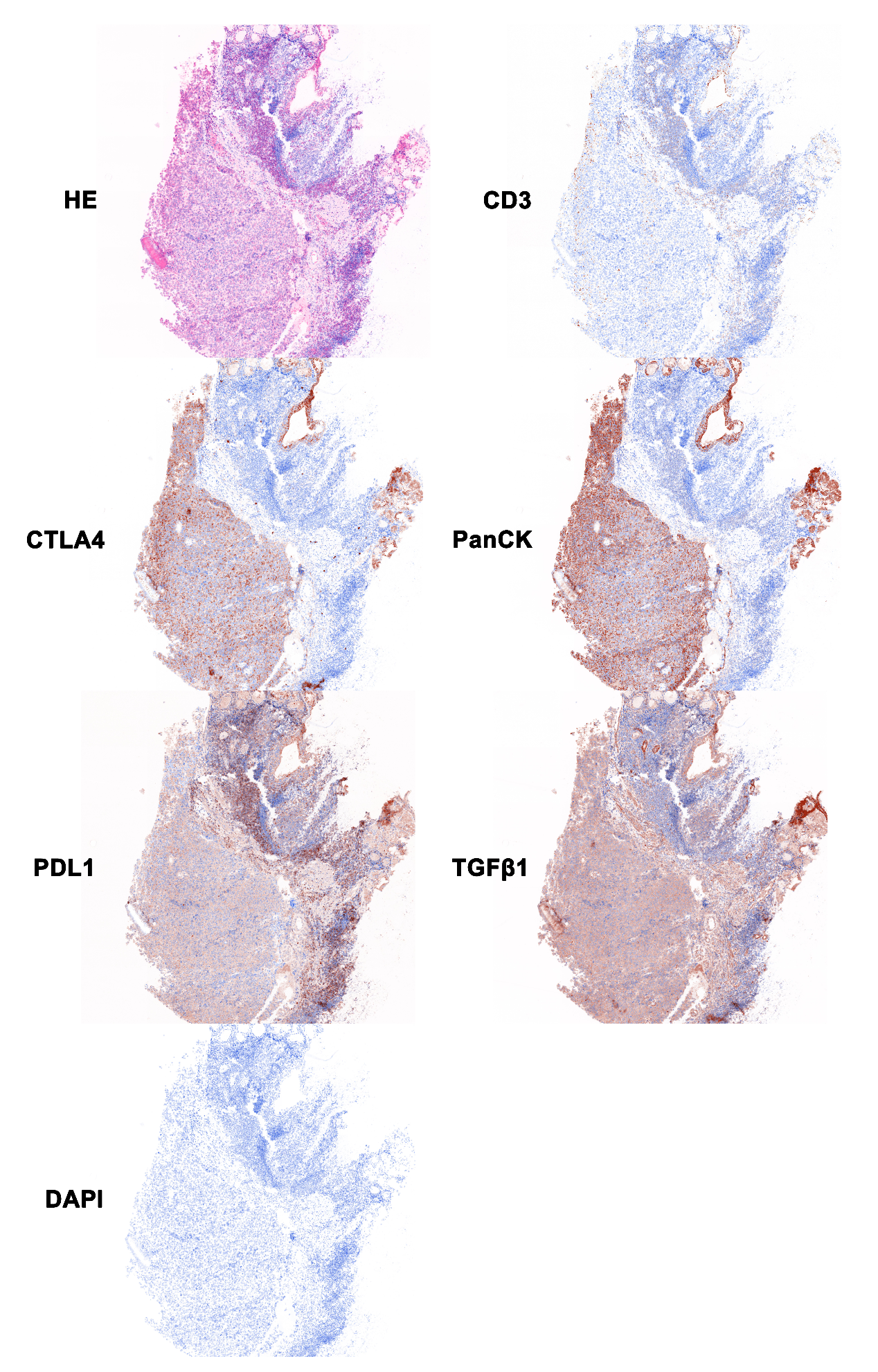


**Supplementary Fig. S2.** Construction of the Risk by the LASSO model. (A) The LASSO-COX regression model was used to generate the prognostic scoring system named Risk in training cohort. (B) In training cohort, 10-time cross -validation via 1-SE criteria, λ = 0.02665483; log(λ): -3.624785.

**
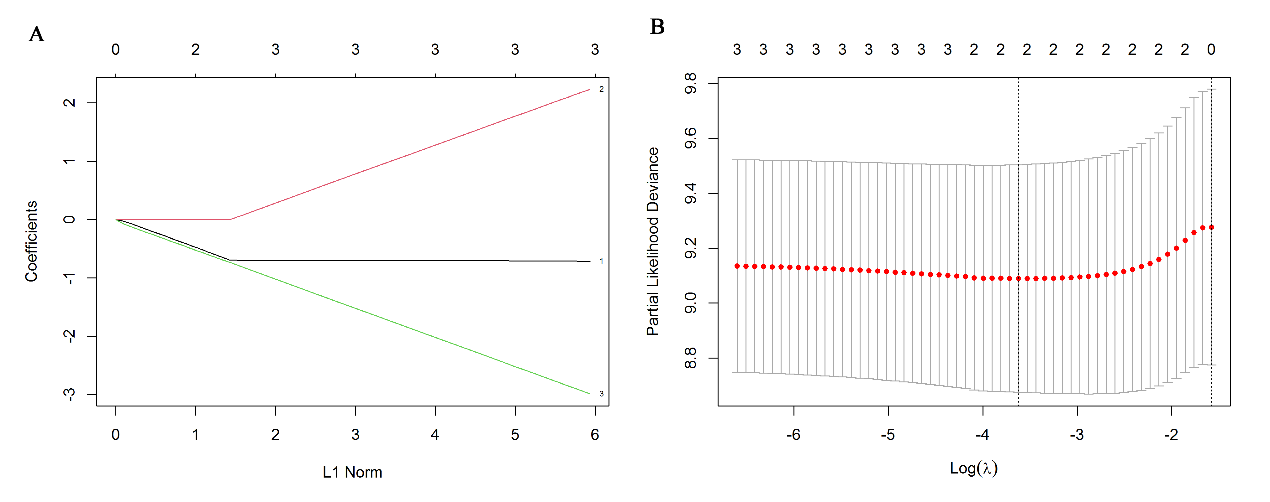
**

**Supplementary Fig. S3.** Risk performance in time-dependent ROC curves and Kaplan–Meier survival analyses in (A) training and (B) validation cohort.

**
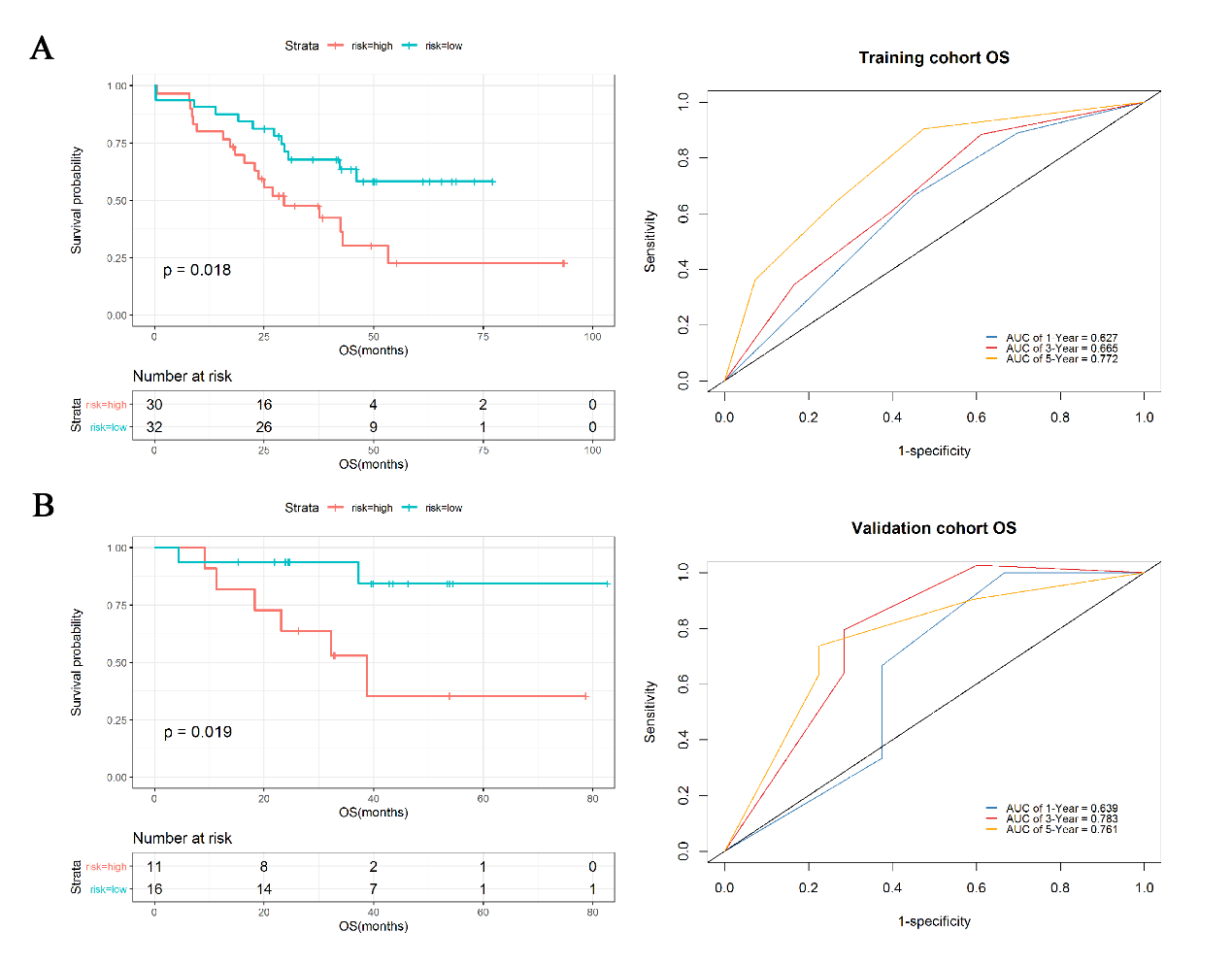
**

**Supplementary Figure S4.** Nomogram for predicting the 1-, 3-, 5-year survival rate for SCLC patients. (A) nomogram. (B) The ROC curve analyses revealed the accuracy of prognosis. (C, D, E) Calibration curves revealed the prediction effect of the Nomogram graph. (F, G) Decision curve analysis showed the net clinical benefit of the nomogram.

**
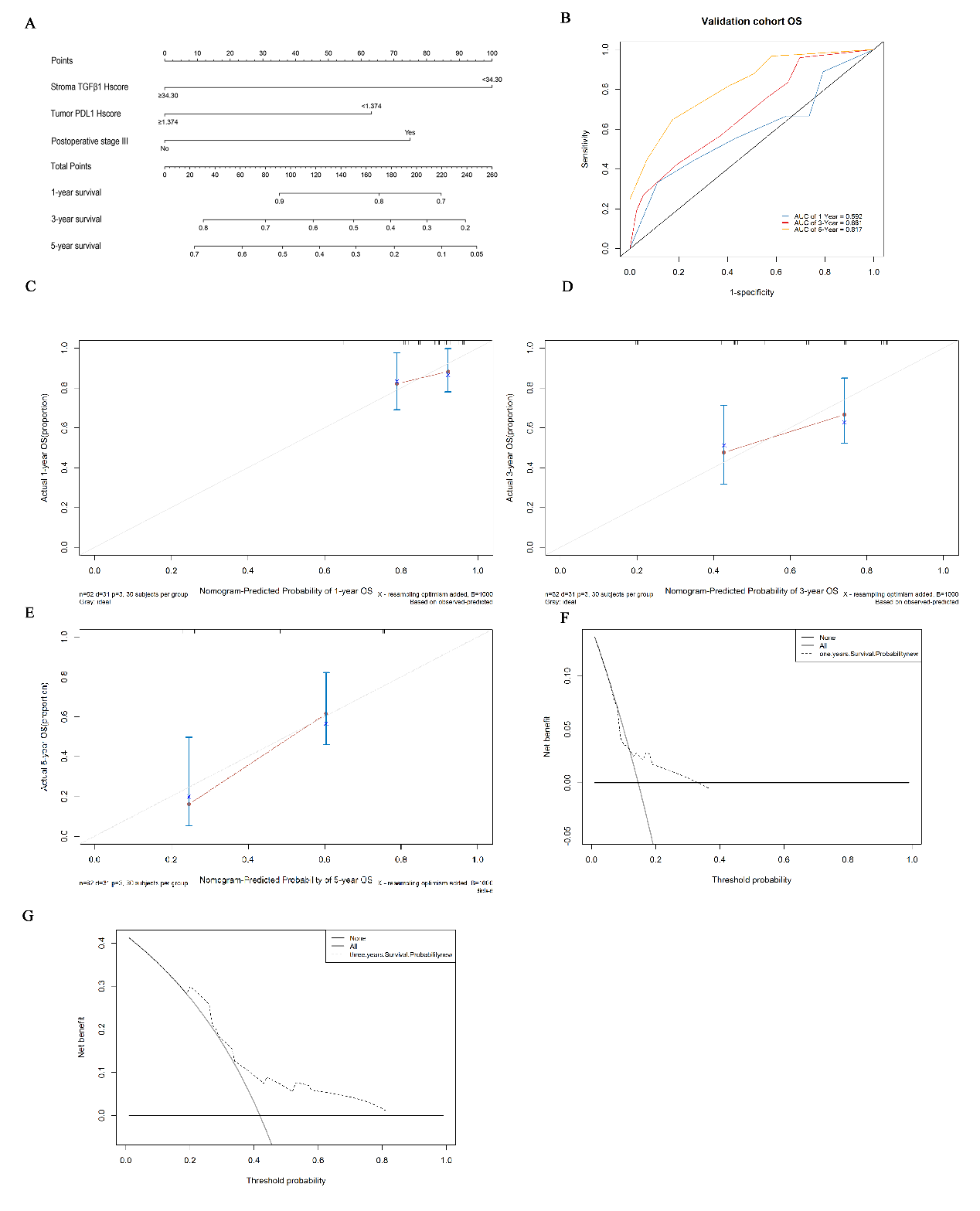
**

**Supplementary Fig. S5.**  Expression of CAF markers in MEF and CAF cells.


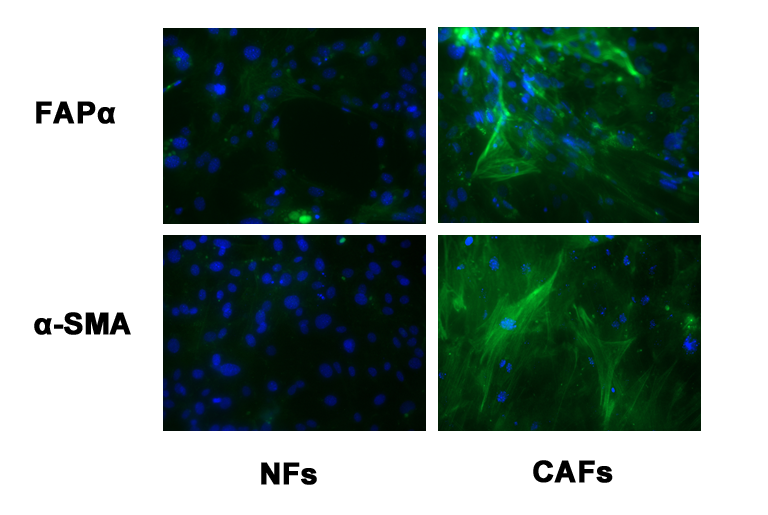


NF: normal fibroblast, CAF: cancer associated fibroblast, α-SMA: alpha-smooth muscle actin, FAPα: fibroblast activation protein-alpha.

**Supplementary Fig. S6.** TGFβ1 was stable knockdown and overexpression in (a) MEF, (b) LLC and (c) HF1 cell lines by recombinant lentiviral infection. The levels of TGFβ1 were examined by western blotting and Elisa test (d). Control: normal cell without recombinant lentiviral infection; shNC and oxNC: control groups corresponding to knockdown and overexpression of TGFβ1 recombinant lentiviral infection; shTGFβ1 and TGFβ1ox: knockdown and overexpression of TGFβ1 recombinant lentiviral infection. Statistical significance is shown (*p < 0.05; **p < 0.01).

**
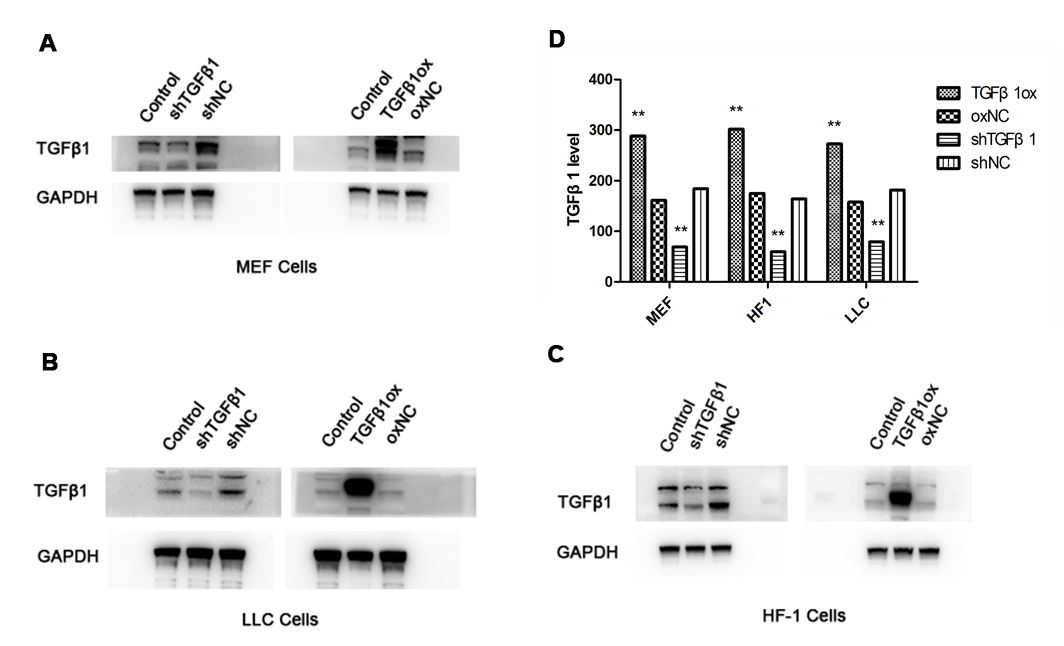
**

MEF: mouse fetal fibroblast cell, LLC: Lewis lung cancer cell, HF-1: human fetal lung fibroblast cell.

Supplementary Fig. S7 TGFβ1 level produced by CAFs affected the apoptosis of lung cancer cells. TGFβ1 overexpression in CAFs led to a significant increase in the expression of PARP1 and cleaved-caspase3, compared with CAFs with TGFβ1 knocked down. LLC: Lewis lung cancer cell, KLN205: mouse squamous cell lung cancer cell, H446: human small cell lung cancer cell


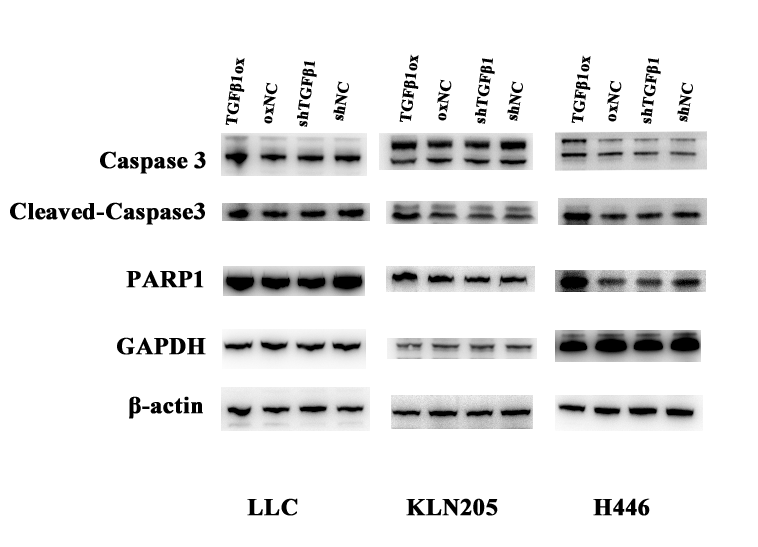

Supplement: Supplementary file 2 [file Table_2.DOCX]
