# Supplementary figures and images for: TGFβ1 in Cancer-Associated Fibroblasts Is Associated With Progression and Radiosensitivity in Small-Cell Lung Cancer
Source: Front Cell Dev Biol. 2021 May 20;9:667645. doi: 10.3389/fcell.2021.667645 (PMC8172974; doi:10.3389/fcell.2021.667645)

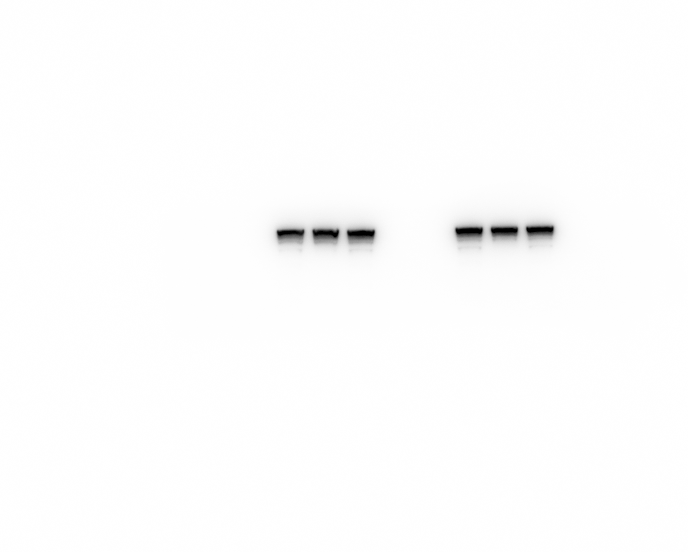

Supplement: Supplementary file 3 [file Data_Sheet_1.ZIP › original images/Supplementary Fig. S6/Supplementary Fig. S6.HF-1.GAPDH.Tif]

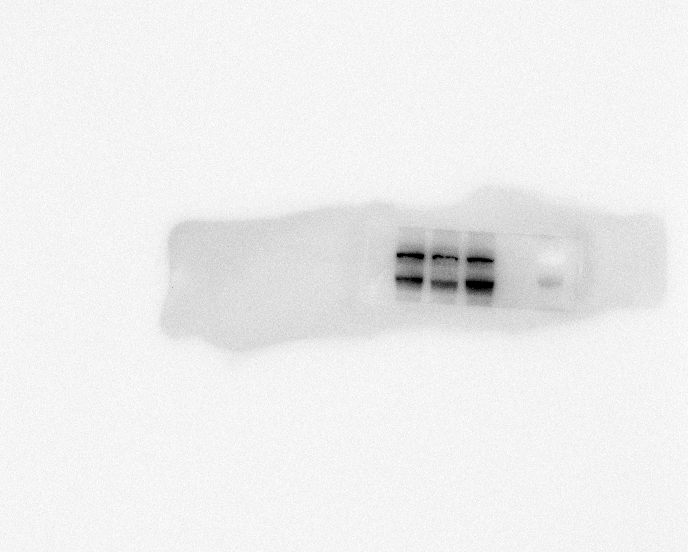

Supplement: Supplementary file 3 [file Data_Sheet_1.ZIP › original images/Supplementary Fig. S6/Supplementary Fig. S6.HF-1.TGFβ1 (2).Tif]

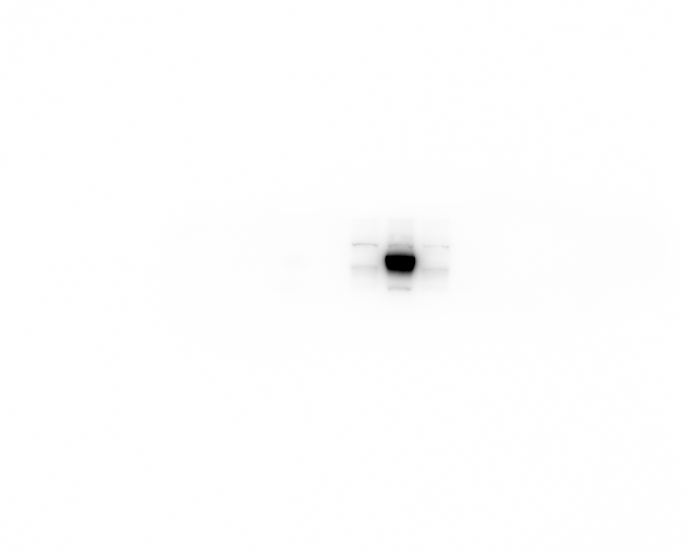

Supplement: Supplementary file 3 [file Data_Sheet_1.ZIP › original images/Supplementary Fig. S6/Supplementary Fig. S6.HF-1.TGFβ1.Tif]

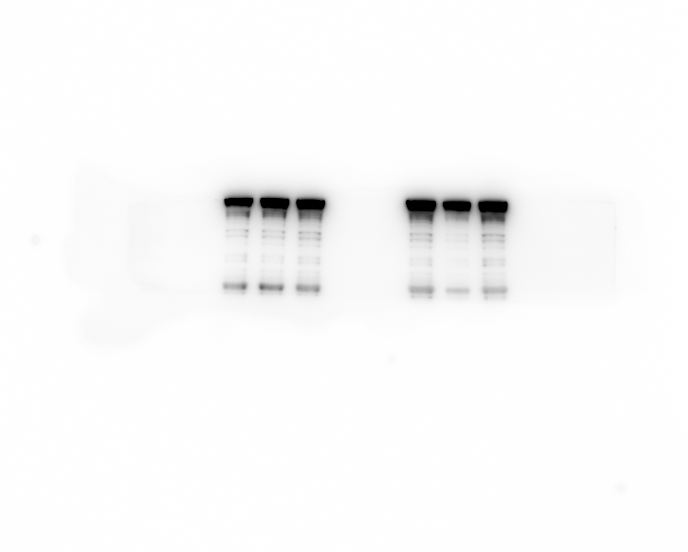

Supplement: Supplementary file 3 [file Data_Sheet_1.ZIP › original images/Supplementary Fig. S6/Supplementary Fig. S6.LLC.GAPDH.Tif]

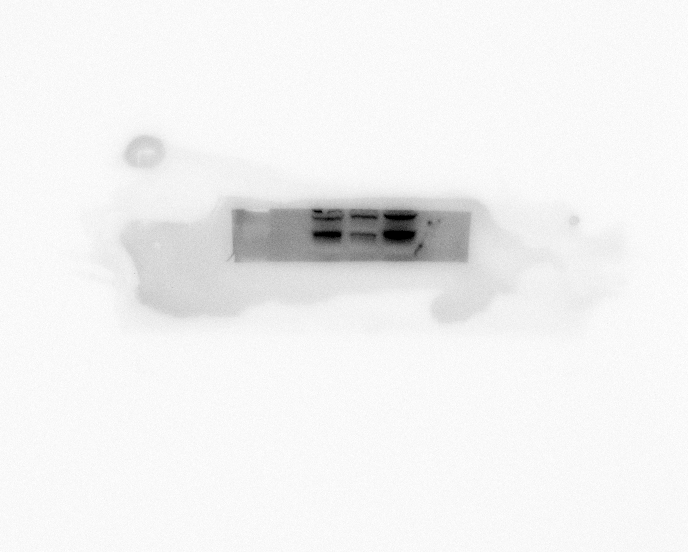

Supplement: Supplementary file 3 [file Data_Sheet_1.ZIP › original images/Supplementary Fig. S6/Supplementary Fig. S6.LLC.TGFβ1(2).Tif]

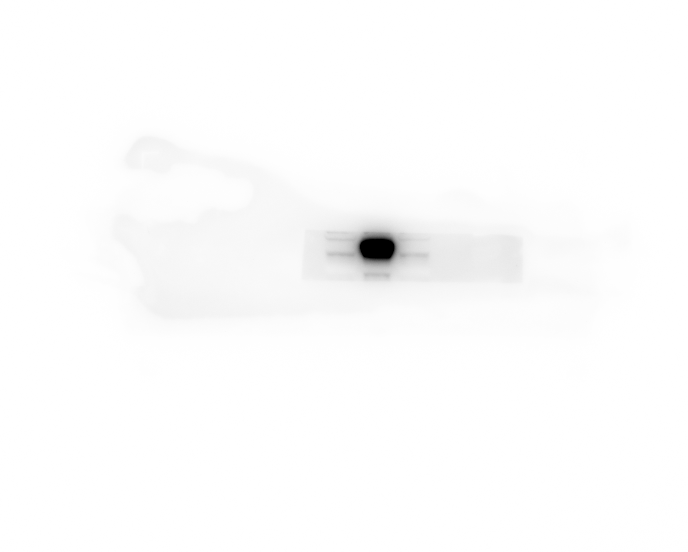

Supplement: Supplementary file 3 [file Data_Sheet_1.ZIP › original images/Supplementary Fig. S6/Supplementary Fig. S6.LLC.TGFβ1.Tif]

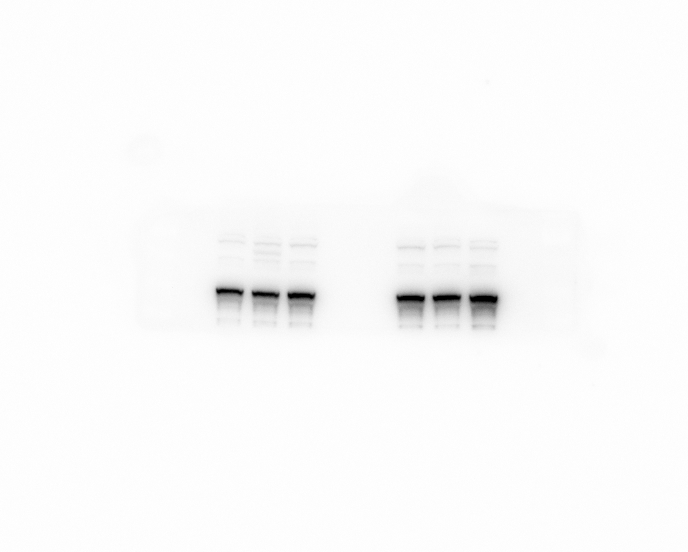

Supplement: Supplementary file 3 [file Data_Sheet_1.ZIP › original images/Supplementary Fig. S6/Supplementary Fig. S6.MEF.GAPDH.Tif]

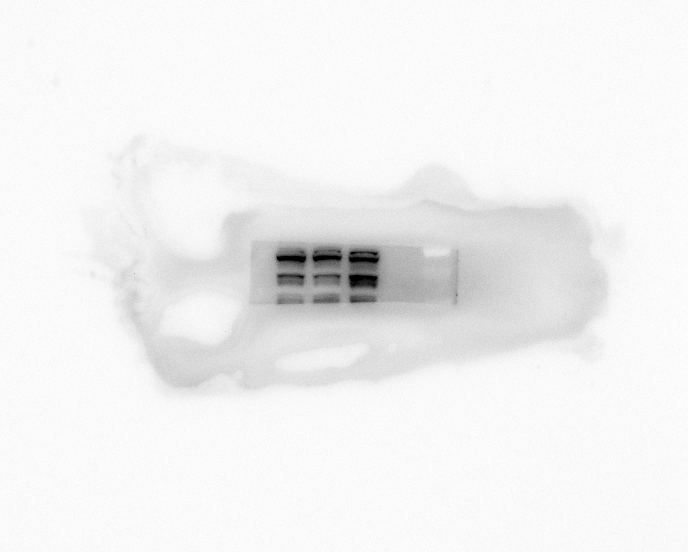

Supplement: Supplementary file 3 [file Data_Sheet_1.ZIP › original images/Supplementary Fig. S6/Supplementary Fig. S6.MEF.TGFβ1(2).Tif]

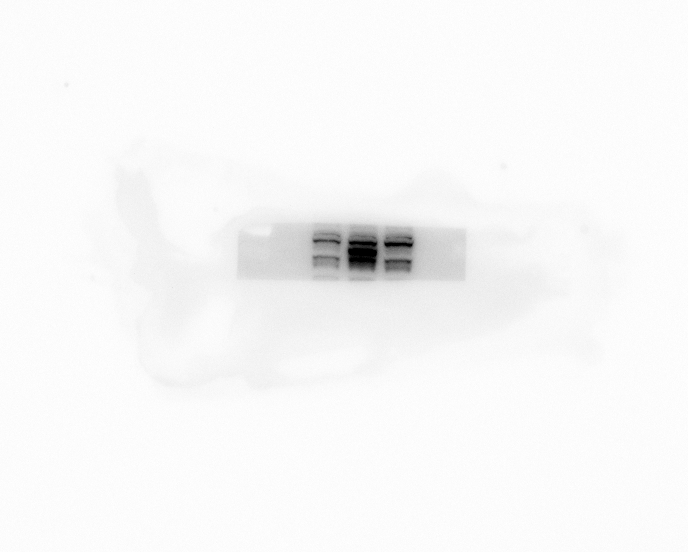

Supplement: Supplementary file 3 [file Data_Sheet_1.ZIP › original images/Supplementary Fig. S6/Supplementary Fig. S6.MEF.TGFβ1.Tif]

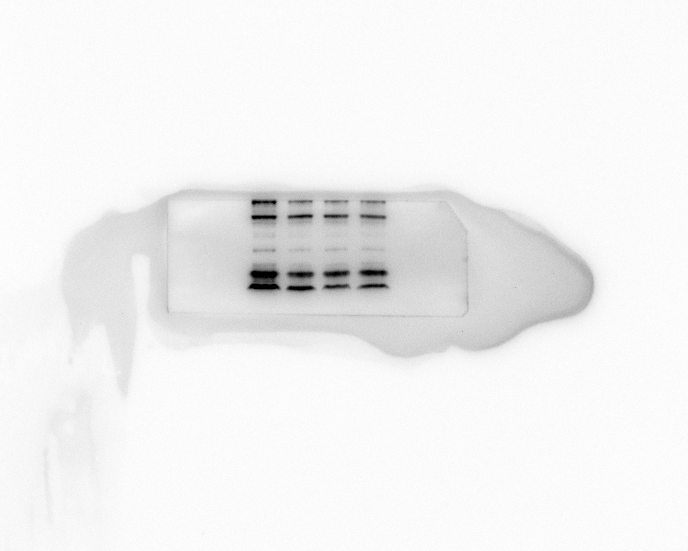

Supplement: Supplementary file 3 [file Data_Sheet_1.ZIP › original images/Supplementary Fig. S7/Supplementary Fig. S7.H446.caspase3.tif]

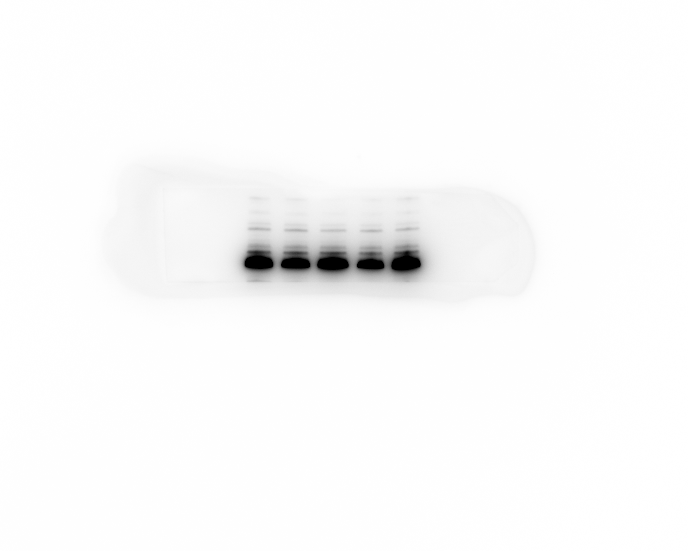

Supplement: Supplementary file 3 [file Data_Sheet_1.ZIP › original images/Supplementary Fig. S7/Supplementary Fig. S7.H446.GAPDH.tif]

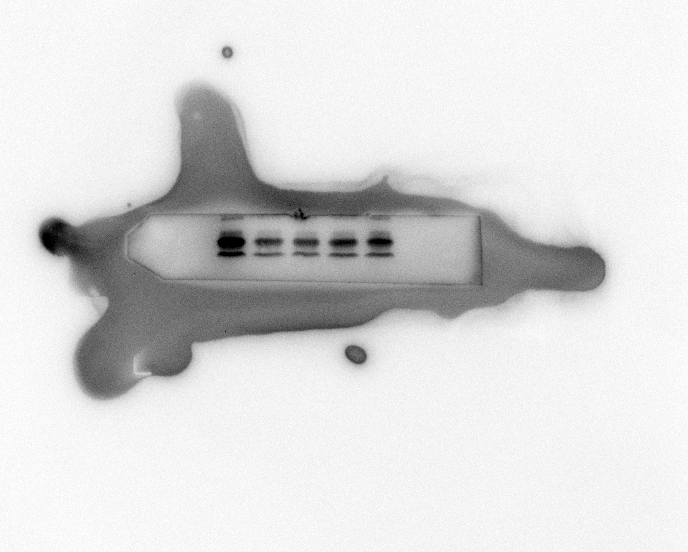

Supplement: Supplementary file 3 [file Data_Sheet_1.ZIP › original images/Supplementary Fig. S7/Supplementary Fig. S7.H446.PARP.Tif]

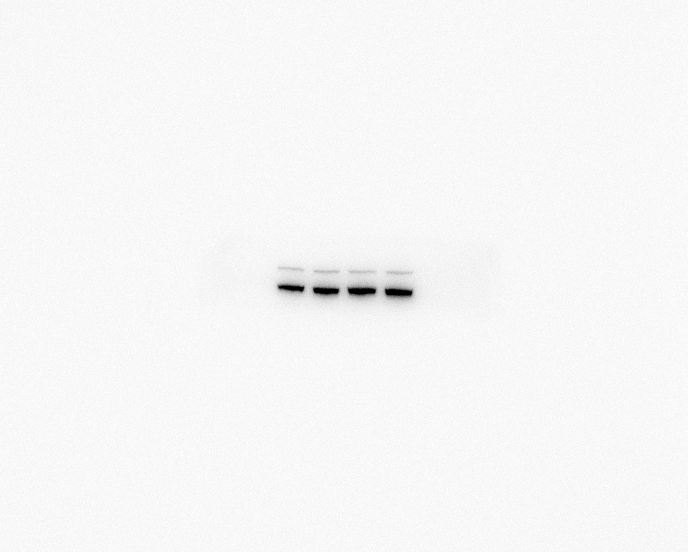

Supplement: Supplementary file 3 [file Data_Sheet_1.ZIP › original images/Supplementary Fig. S7/Supplementary Fig. S7.H446.βactin.Tif]

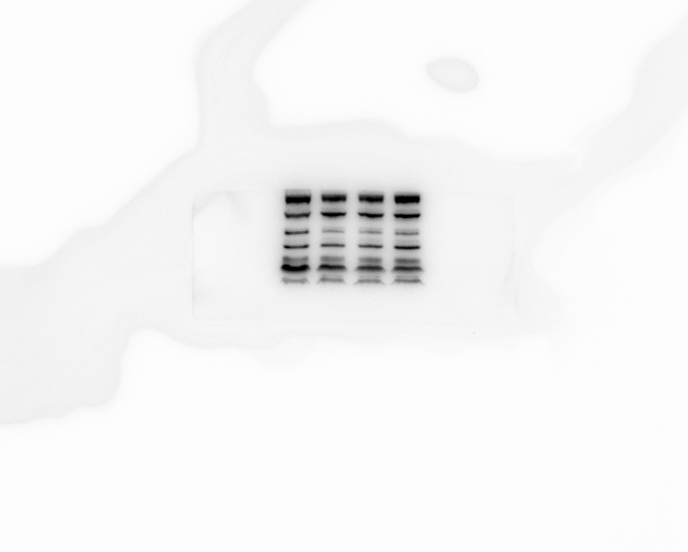

Supplement: Supplementary file 3 [file Data_Sheet_1.ZIP › original images/Supplementary Fig. S7/Supplementary Fig. S7.KLN205.caspase3.Tif]

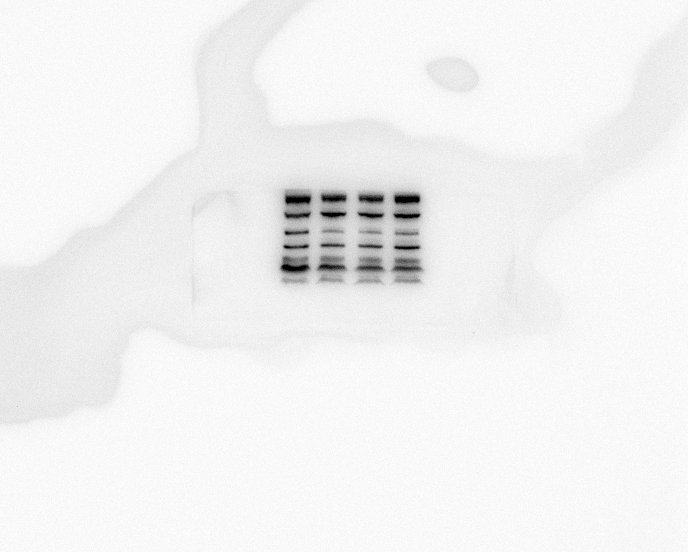

Supplement: Supplementary file 3 [file Data_Sheet_1.ZIP › original images/Supplementary Fig. S7/Supplementary Fig. S7.KLN205.cleaved-caspase3.Tif]

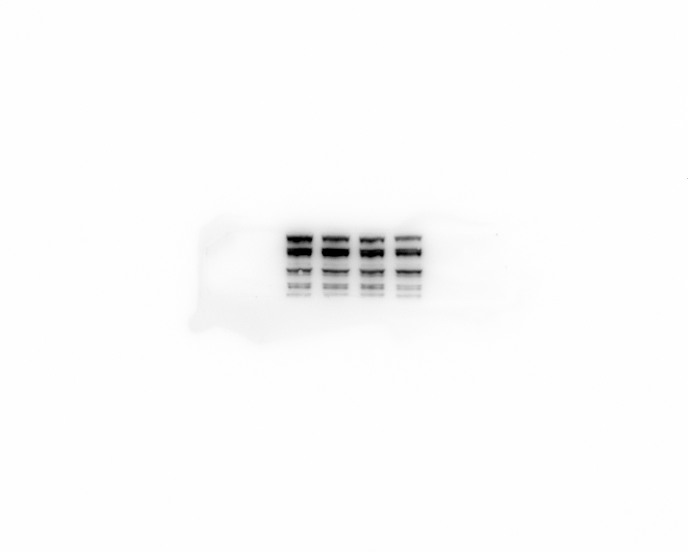

Supplement: Supplementary file 3 [file Data_Sheet_1.ZIP › original images/Supplementary Fig. S7/Supplementary Fig. S7.KLN205.GAPDH.Tif]

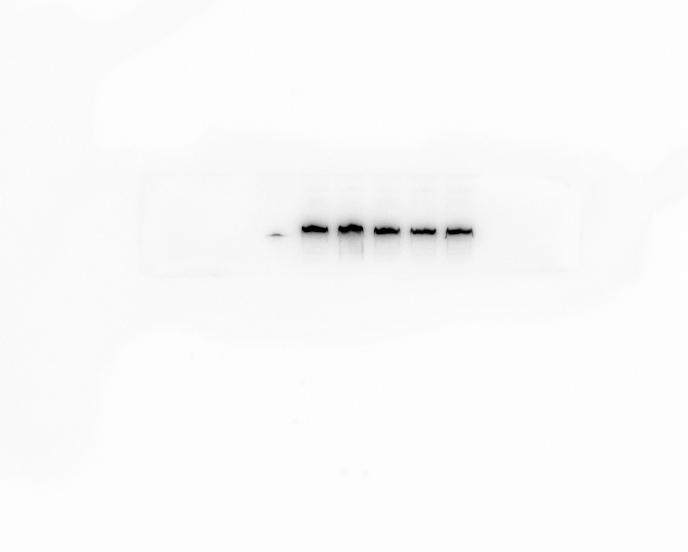

Supplement: Supplementary file 3 [file Data_Sheet_1.ZIP › original images/Supplementary Fig. S7/Supplementary Fig. S7.KLN205.prap.Tif]

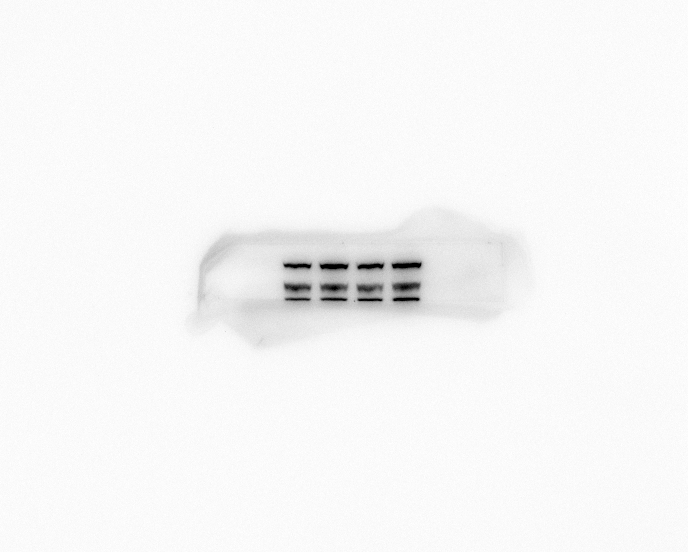

Supplement: Supplementary file 3 [file Data_Sheet_1.ZIP › original images/Supplementary Fig. S7/Supplementary Fig. S7.KLN205.βactin.Tif]

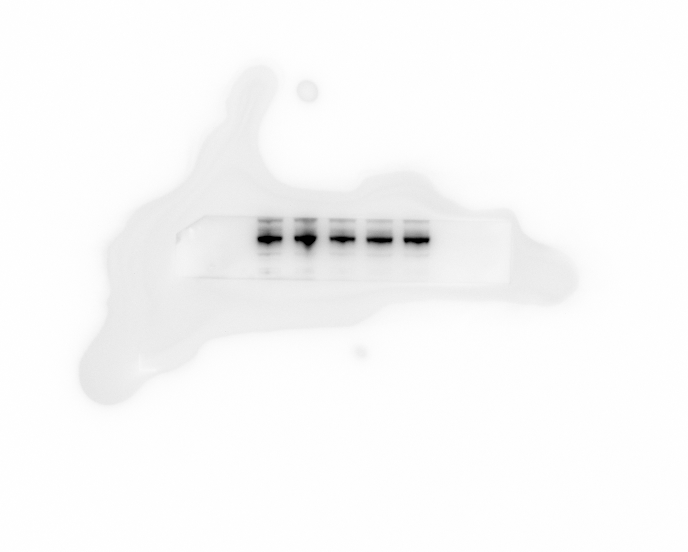

Supplement: Supplementary file 3 [file Data_Sheet_1.ZIP › original images/Supplementary Fig. S7/Supplementary Fig. S7.LLC.caspase3.Tif]

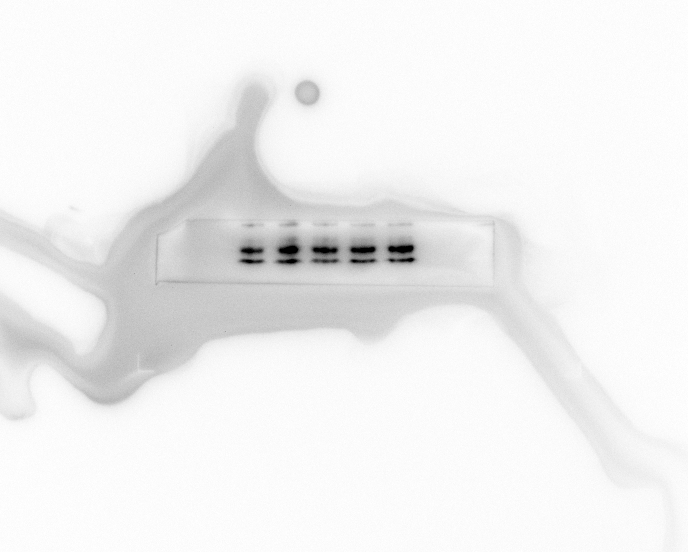

Supplement: Supplementary file 3 [file Data_Sheet_1.ZIP › original images/Supplementary Fig. S7/Supplementary Fig. S7.LLC.cleaved-caspase3.Tif]

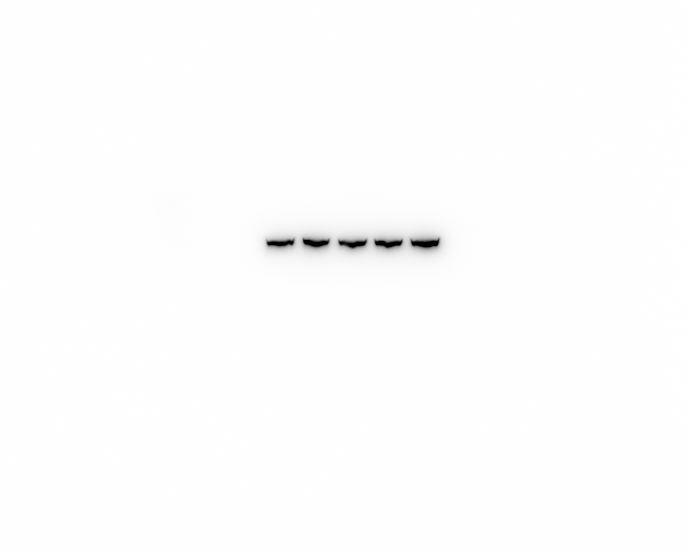

Supplement: Supplementary file 3 [file Data_Sheet_1.ZIP › original images/Supplementary Fig. S7/Supplementary Fig. S7.LLC.GAPDH.Tif]

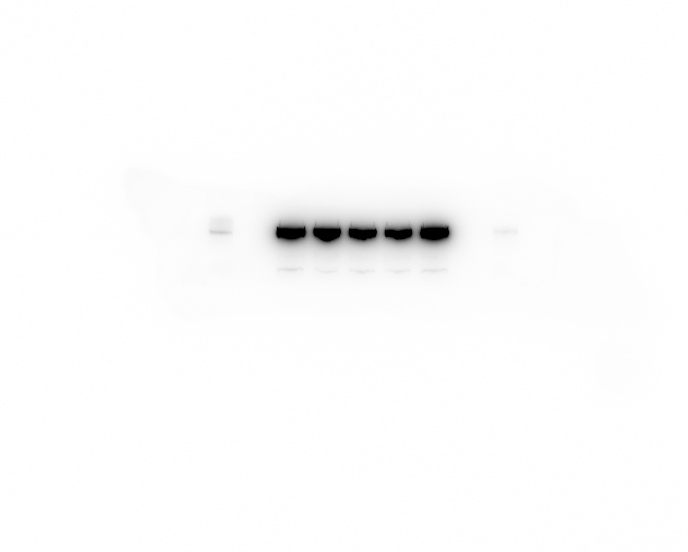

Supplement: Supplementary file 3 [file Data_Sheet_1.ZIP › original images/Supplementary Fig. S7/Supplementary Fig. S7.LLC.PRAP.Tif]

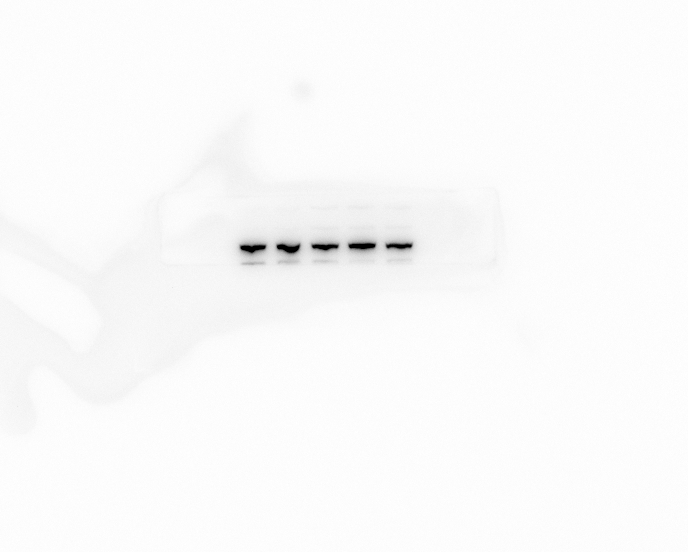

Supplement: Supplementary file 3 [file Data_Sheet_1.ZIP › original images/Supplementary Fig. S7/Supplementary Fig. S7.LLC.βactin.Tif]

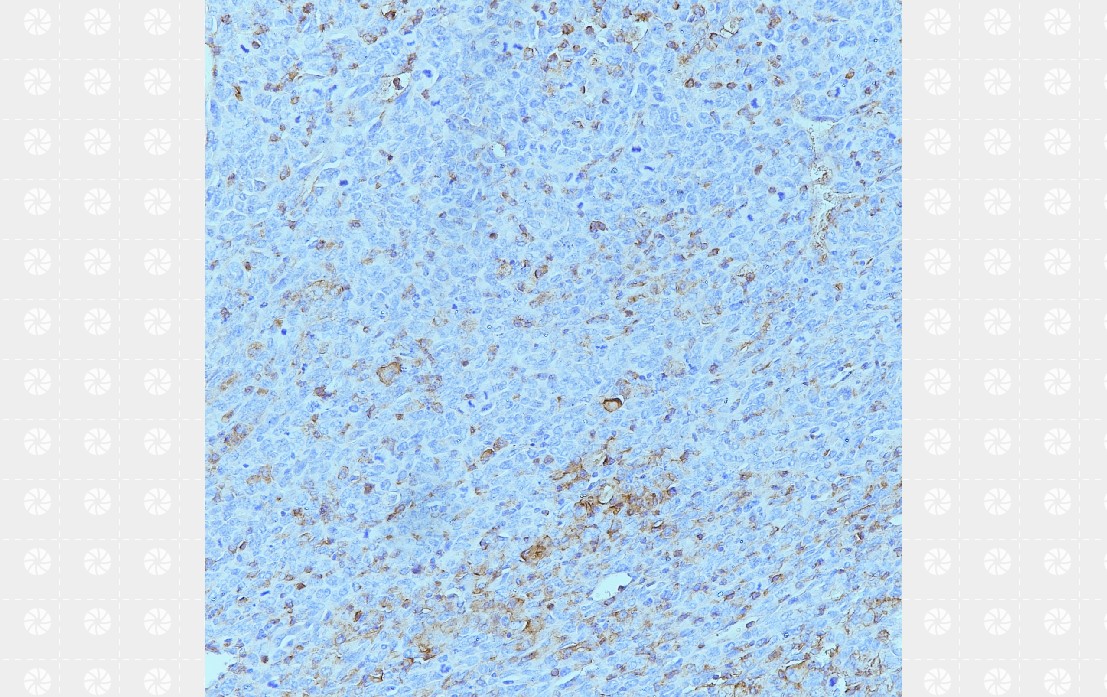

Supplement: Supplementary file 4 [file Data_Sheet_2.ZIP › figure 6 original images/oxNC.CD11b.jpg]

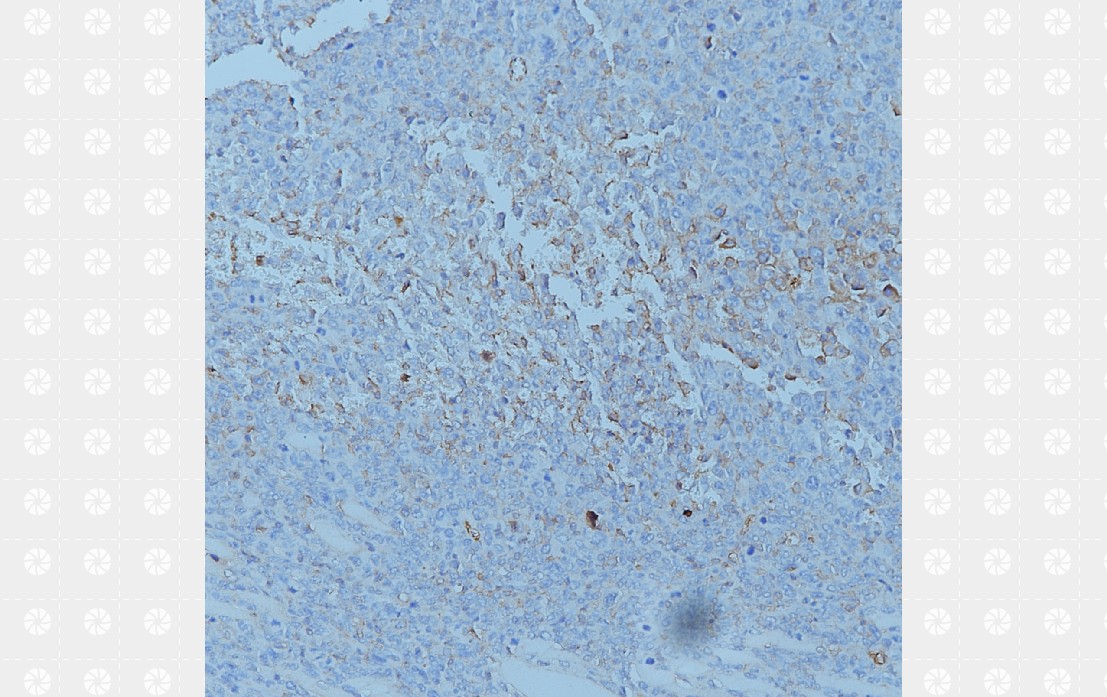

Supplement: Supplementary file 4 [file Data_Sheet_2.ZIP › figure 6 original images/oxNC.CD206.jpg]

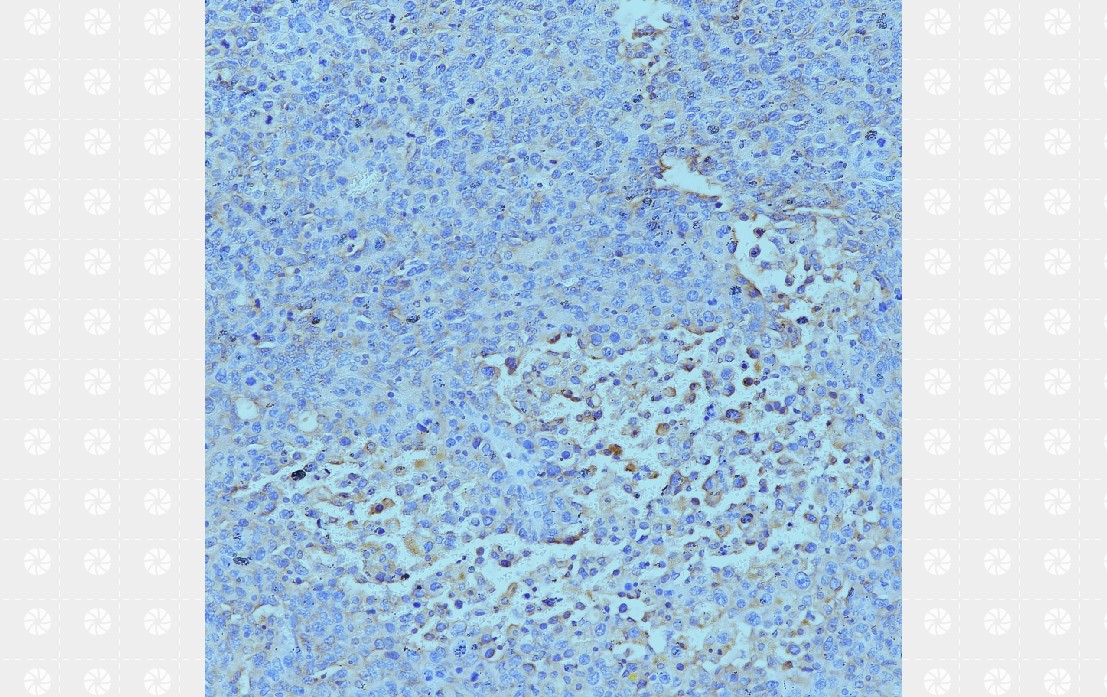

Supplement: Supplementary file 4 [file Data_Sheet_2.ZIP › figure 6 original images/oxNC.CD56.jpg]

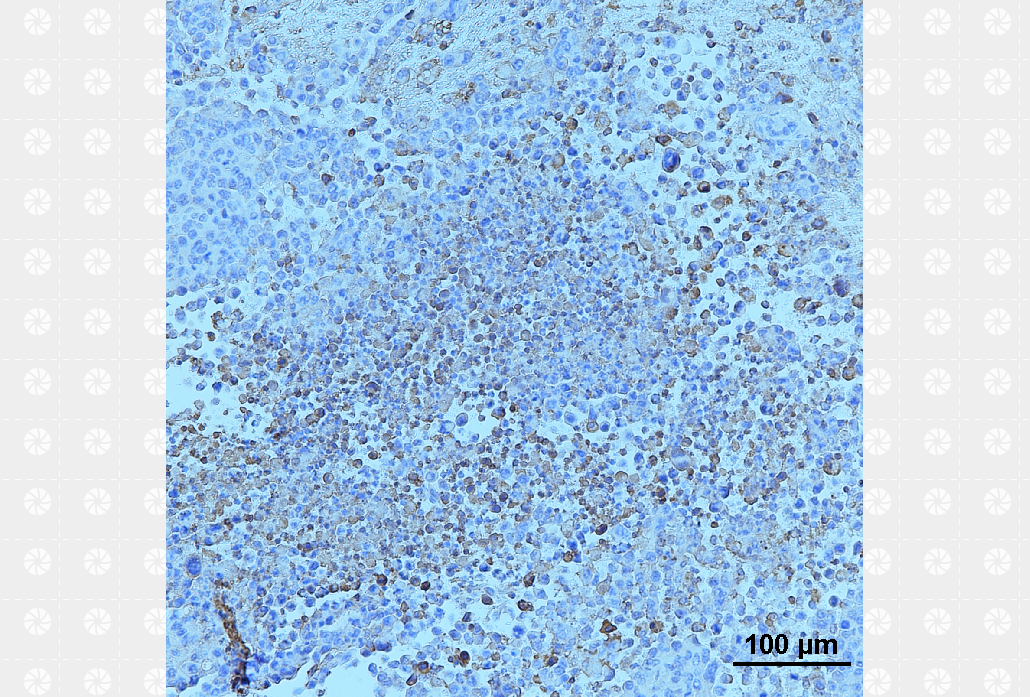

Supplement: Supplementary file 4 [file Data_Sheet_2.ZIP › figure 6 original images/oxNC.CD8.tif]

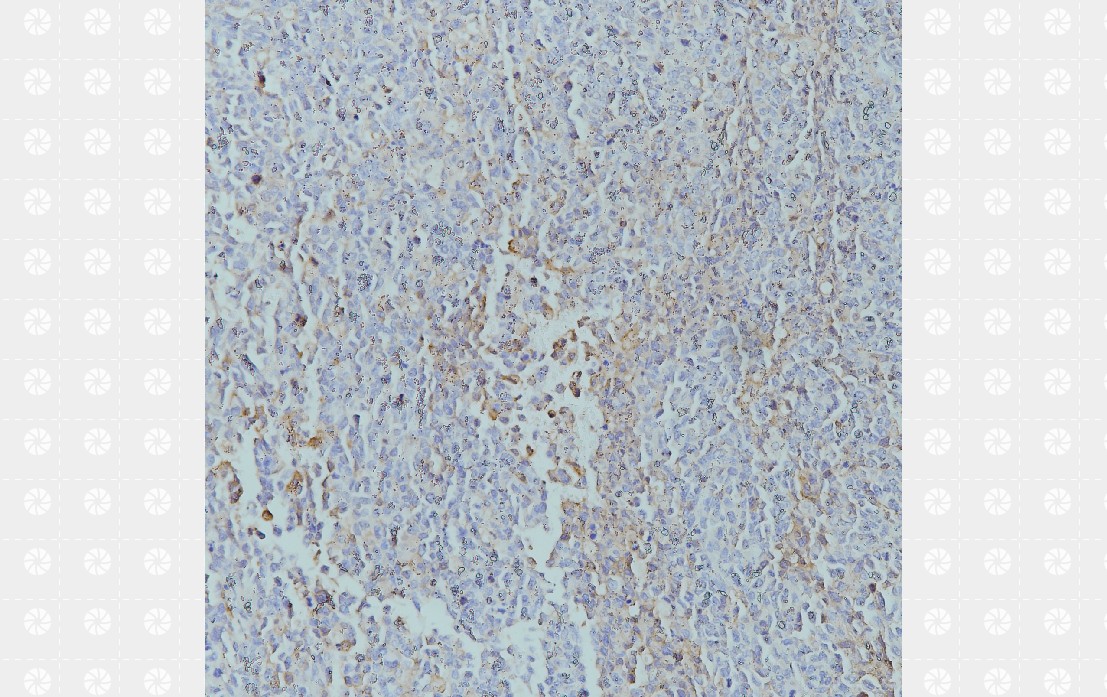

Supplement: Supplementary file 4 [file Data_Sheet_2.ZIP › figure 6 original images/oxNC.CD86.jpg]

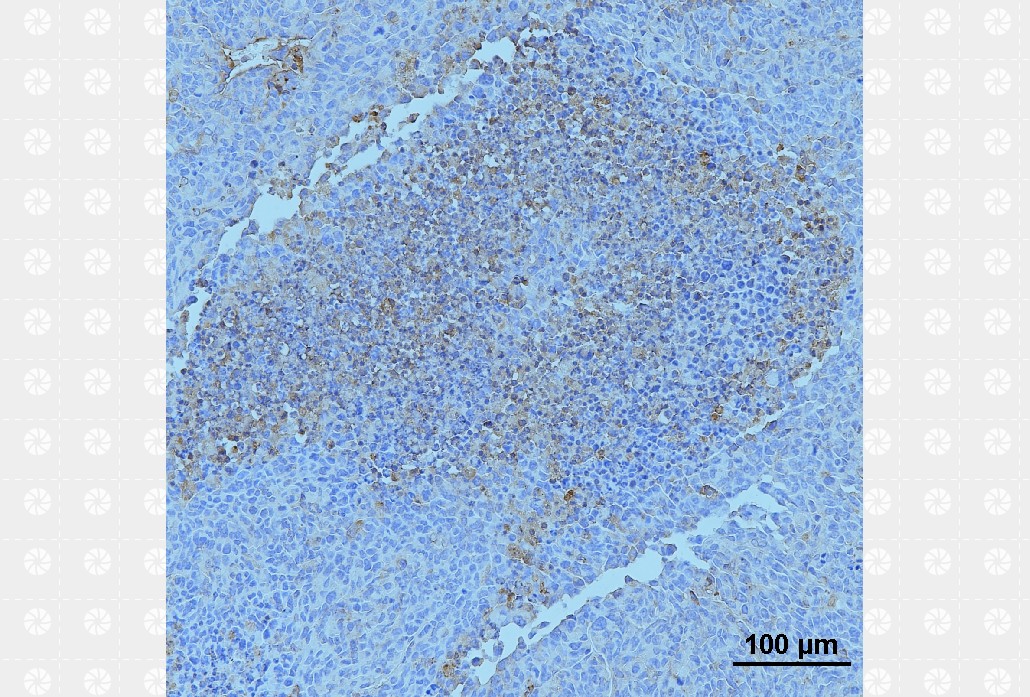

Supplement: Supplementary file 4 [file Data_Sheet_2.ZIP › figure 6 original images/oxNC.FOXP3.jpg]

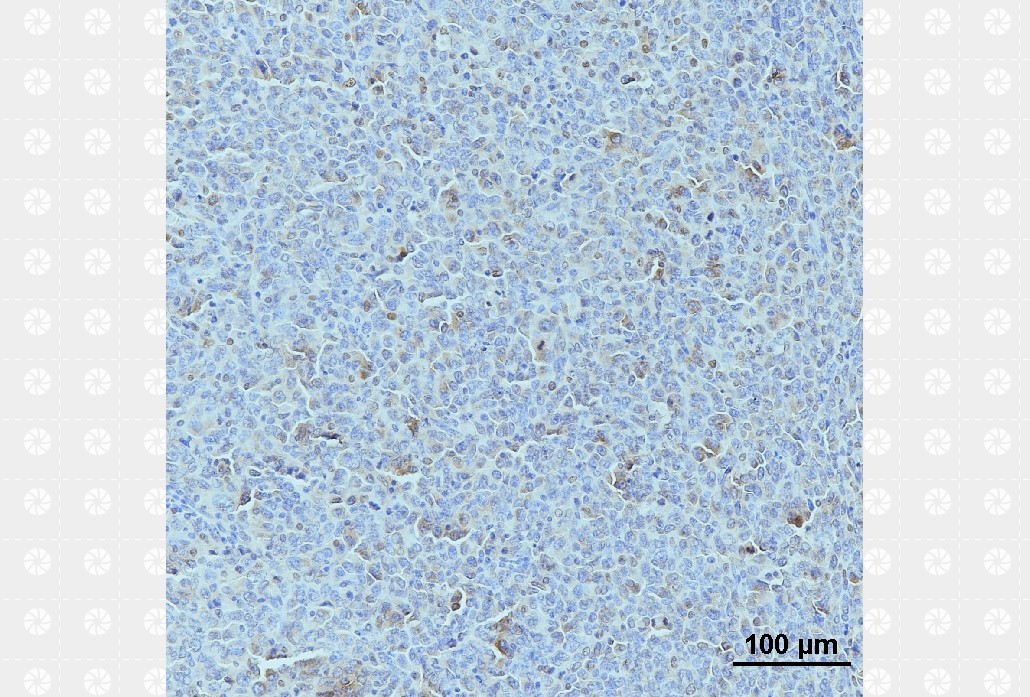

Supplement: Supplementary file 4 [file Data_Sheet_2.ZIP › figure 6 original images/oxNC.PDL1.jpg]

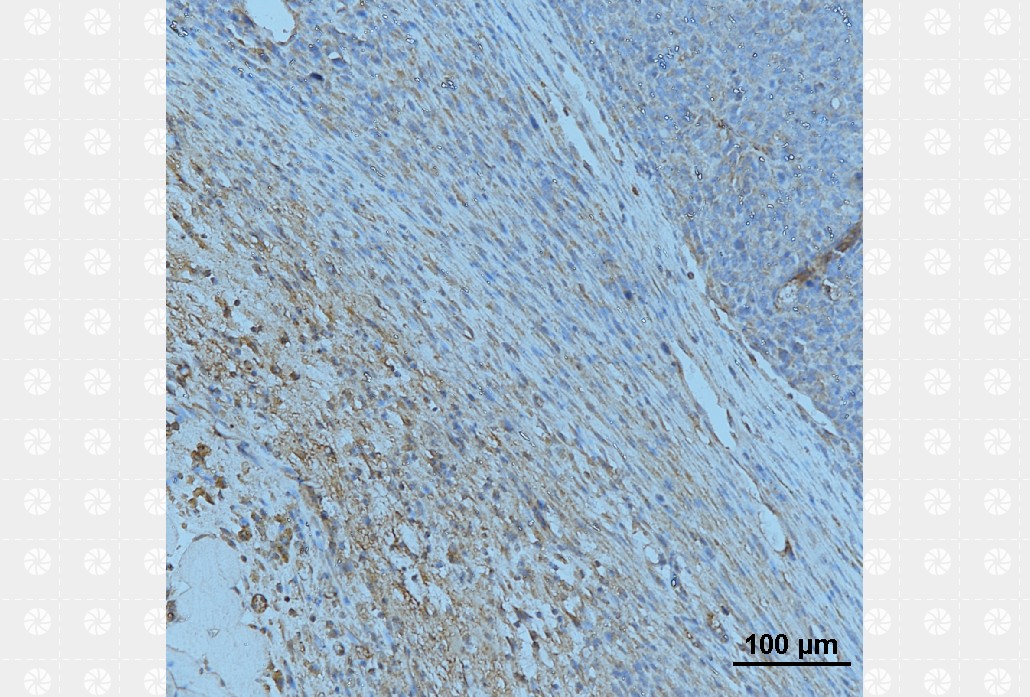

Supplement: Supplementary file 4 [file Data_Sheet_2.ZIP › figure 6 original images/oxNC.TGFβ1.jpg]

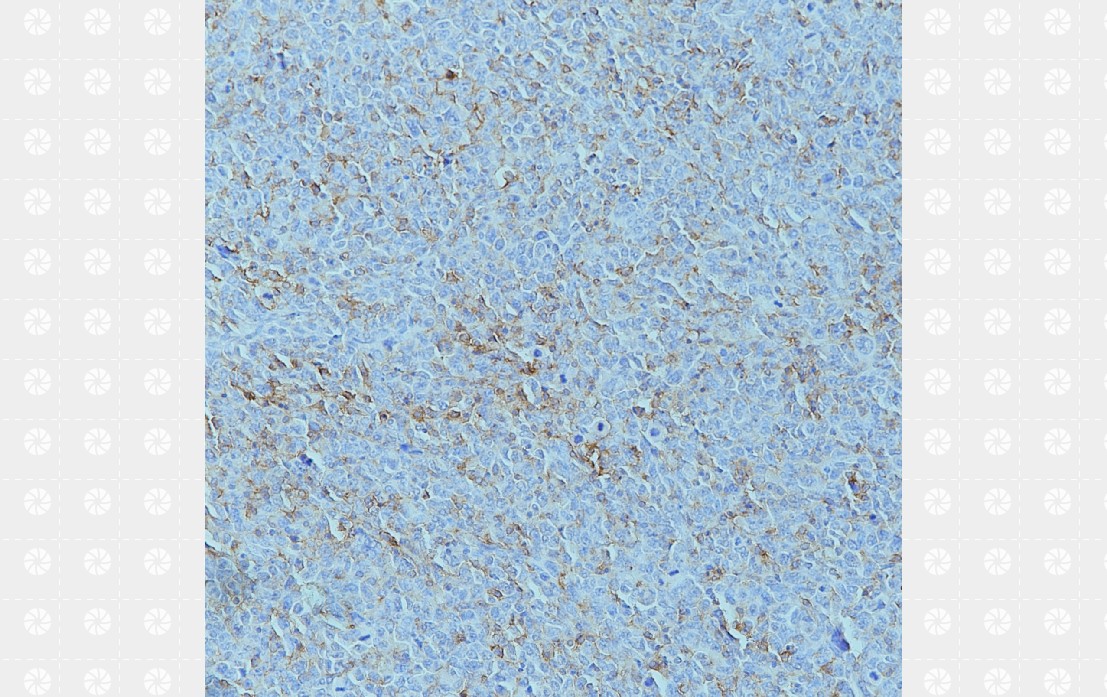

Supplement: Supplementary file 4 [file Data_Sheet_2.ZIP › figure 6 original images/shNC.CD11b.jpg]

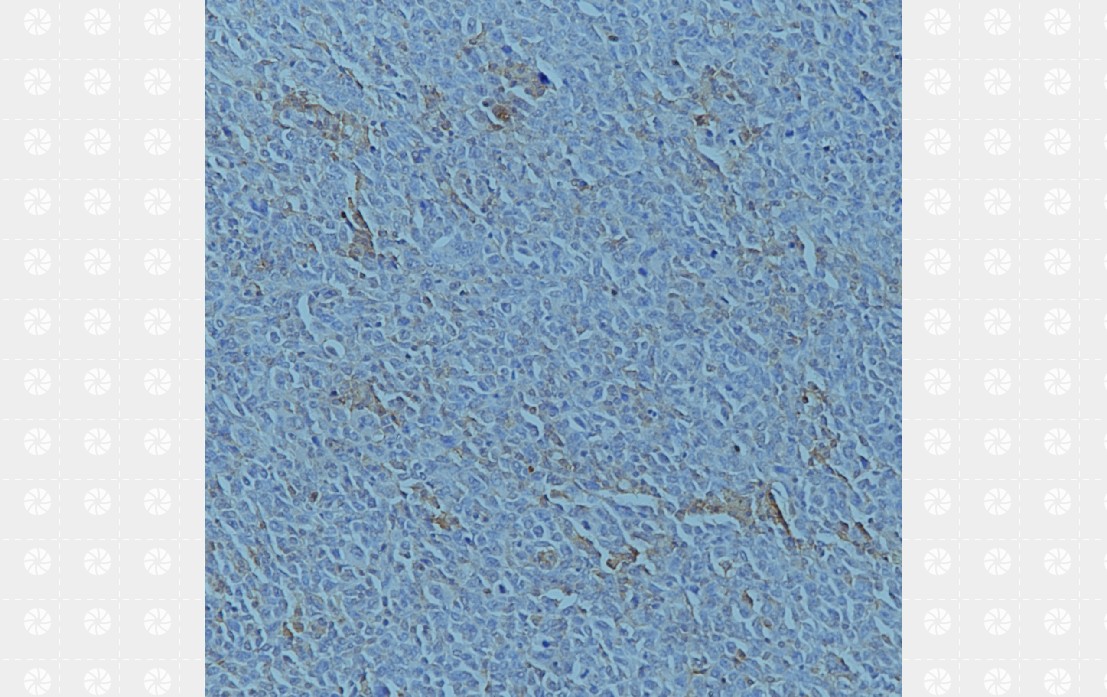

Supplement: Supplementary file 4 [file Data_Sheet_2.ZIP › figure 6 original images/shNC.CD206.jpg]

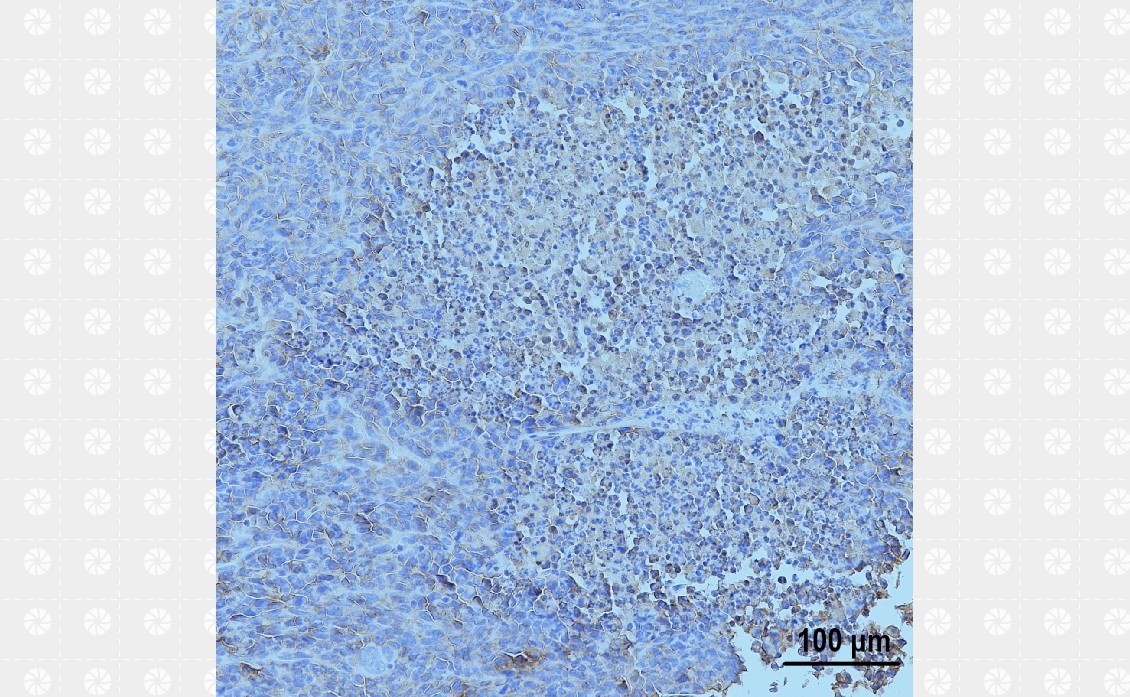

Supplement: Supplementary file 4 [file Data_Sheet_2.ZIP › figure 6 original images/shNC.CD56.jpg]

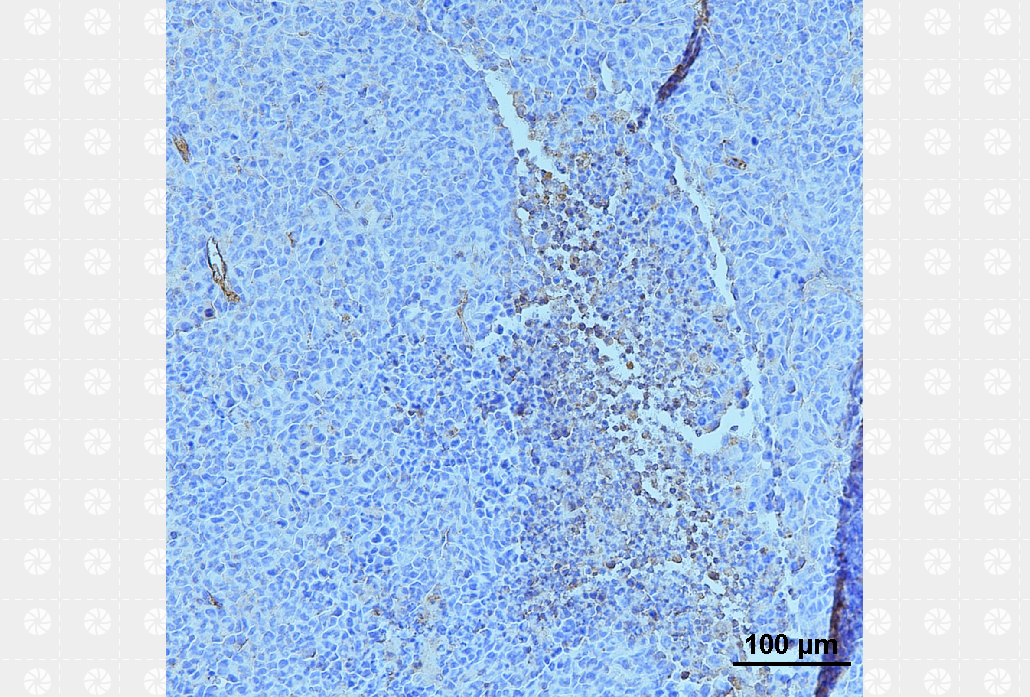

Supplement: Supplementary file 4 [file Data_Sheet_2.ZIP › figure 6 original images/shNC.CD8.tif]

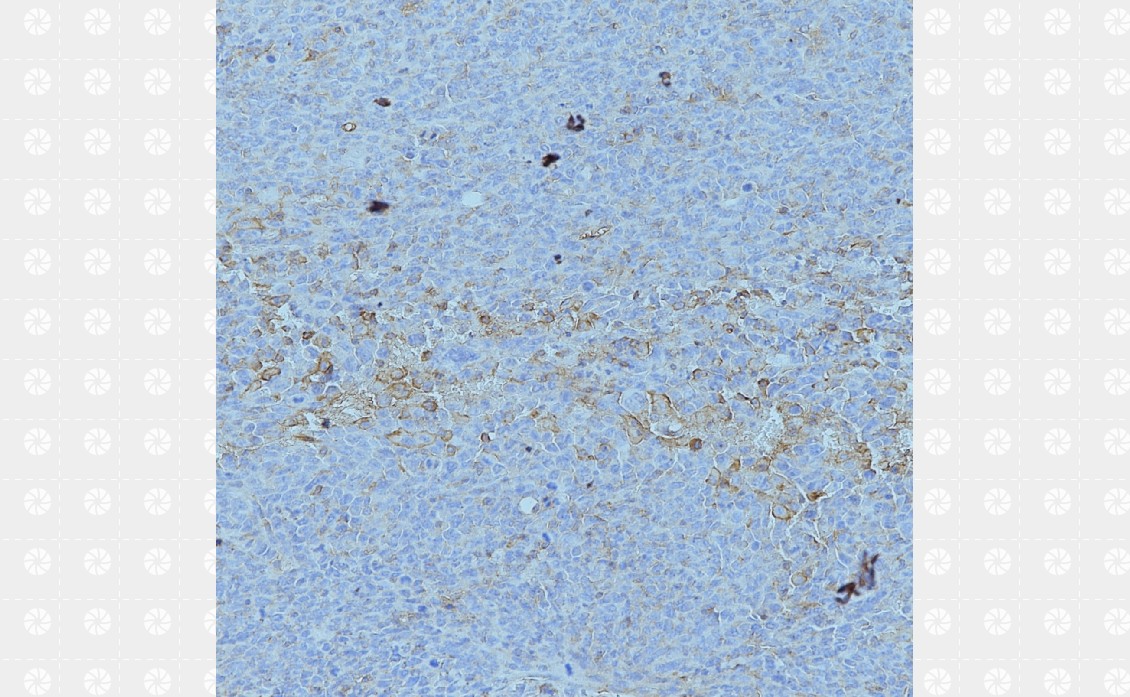

Supplement: Supplementary file 4 [file Data_Sheet_2.ZIP › figure 6 original images/shNC.CD86.jpg]

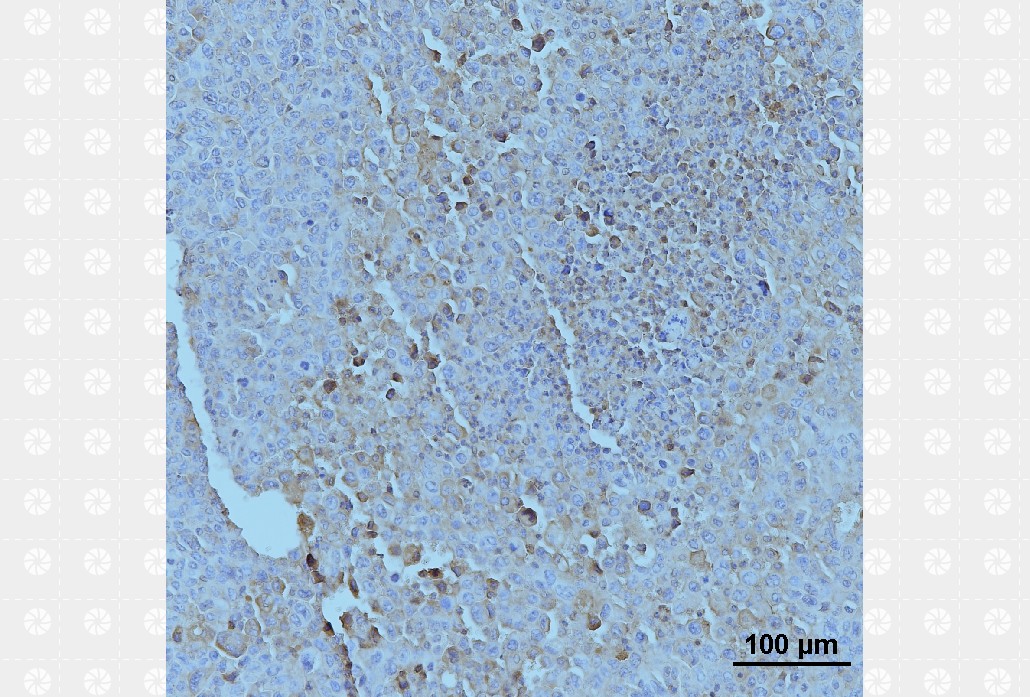

Supplement: Supplementary file 4 [file Data_Sheet_2.ZIP › figure 6 original images/shNC.FOXP3.jpg]

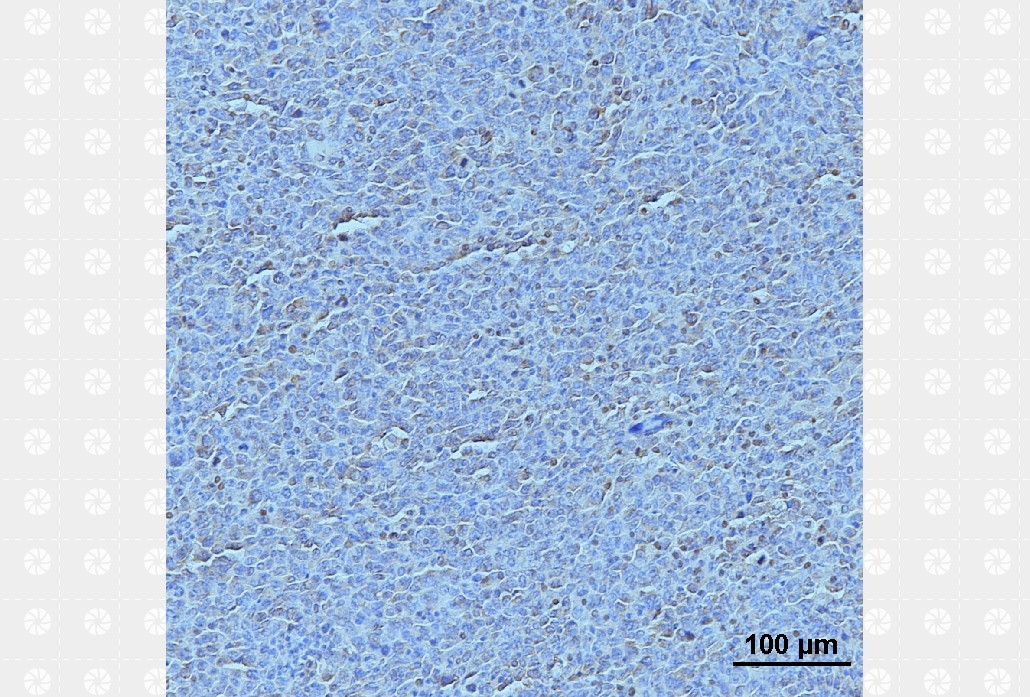

Supplement: Supplementary file 4 [file Data_Sheet_2.ZIP › figure 6 original images/shNC.PDL1.jpg]

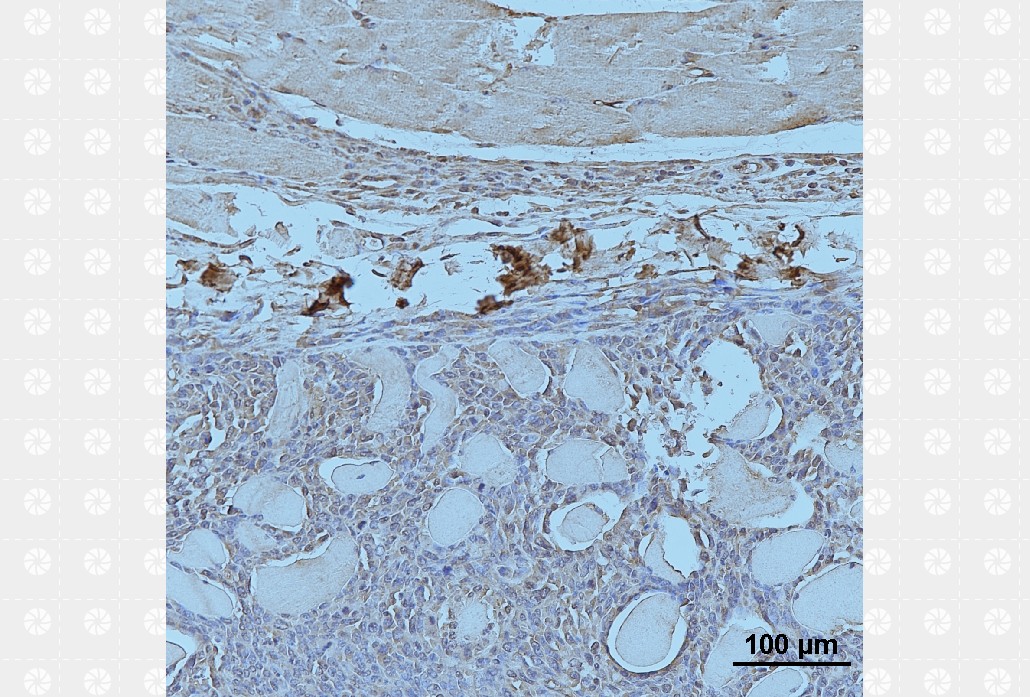

Supplement: Supplementary file 4 [file Data_Sheet_2.ZIP › figure 6 original images/shNC.TGFβ1.jpg]

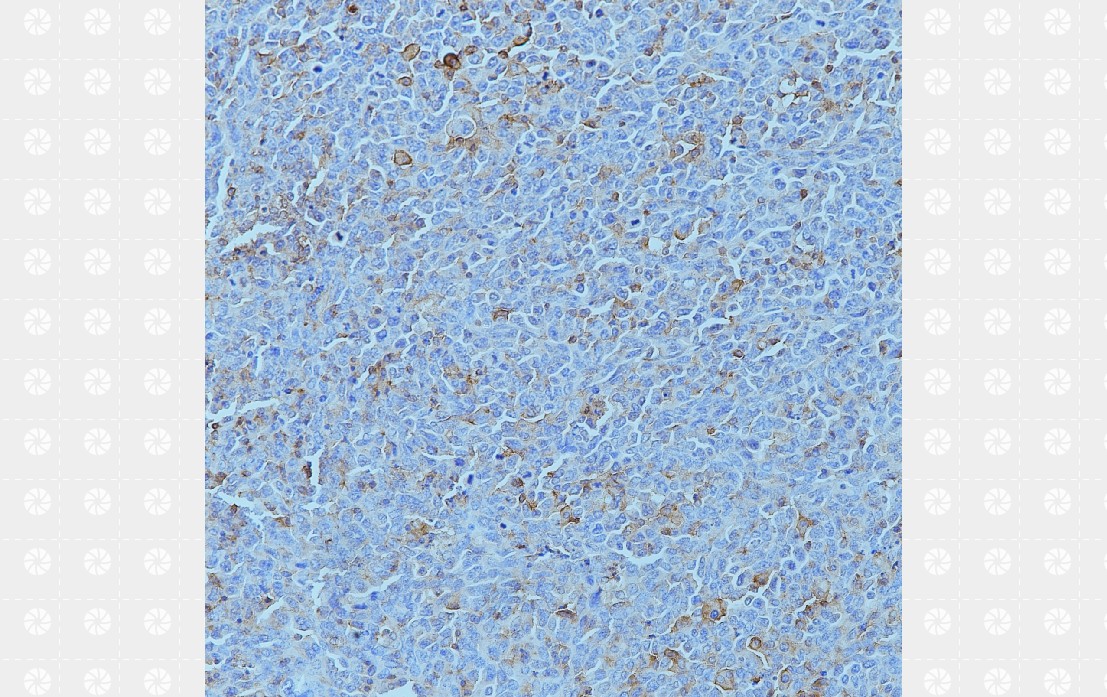

Supplement: Supplementary file 4 [file Data_Sheet_2.ZIP › figure 6 original images/shTGFβ1.CD11b.jpg]

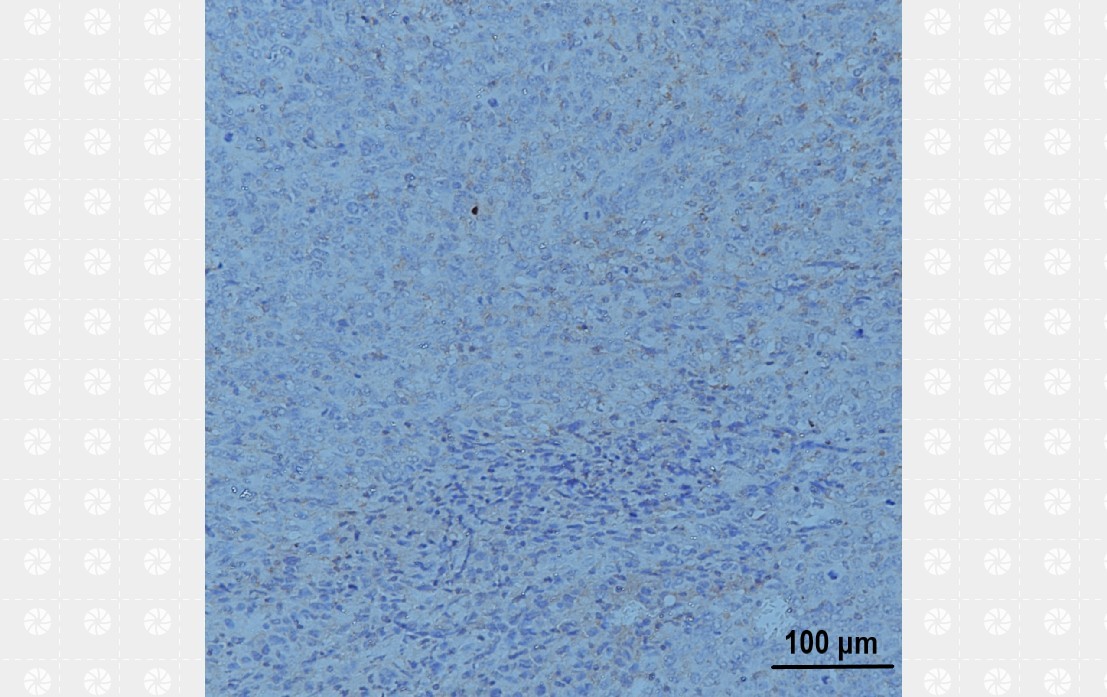

Supplement: Supplementary file 4 [file Data_Sheet_2.ZIP › figure 6 original images/shTGFβ1.CD206.jpg]

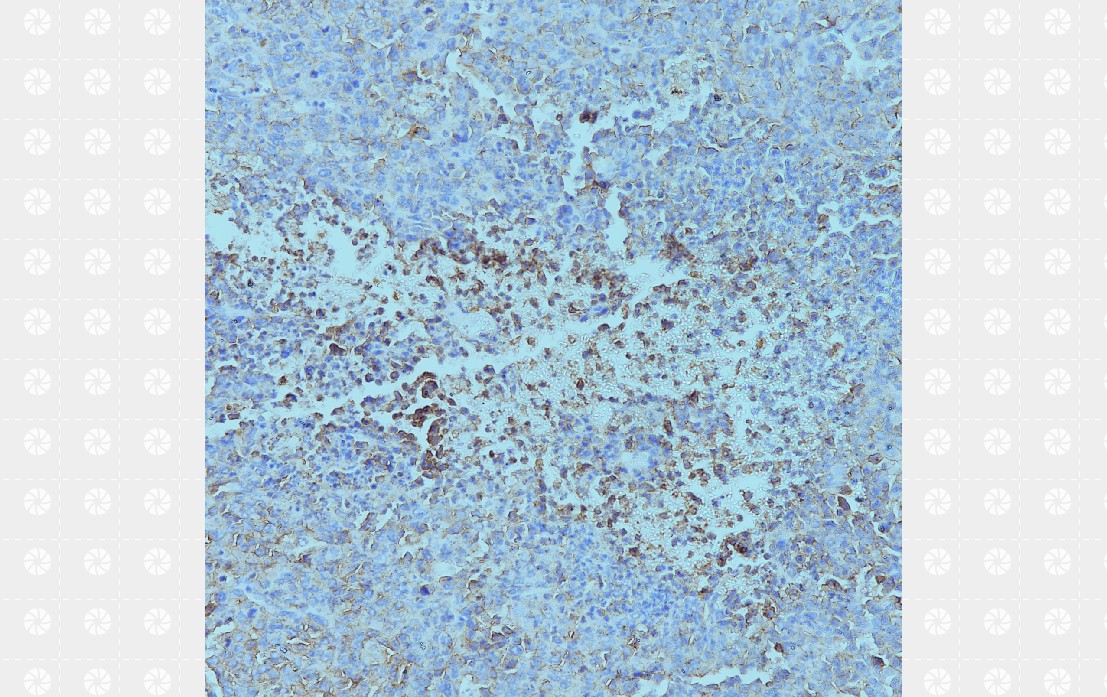

Supplement: Supplementary file 4 [file Data_Sheet_2.ZIP › figure 6 original images/shTGFβ1.CD56.jpg]

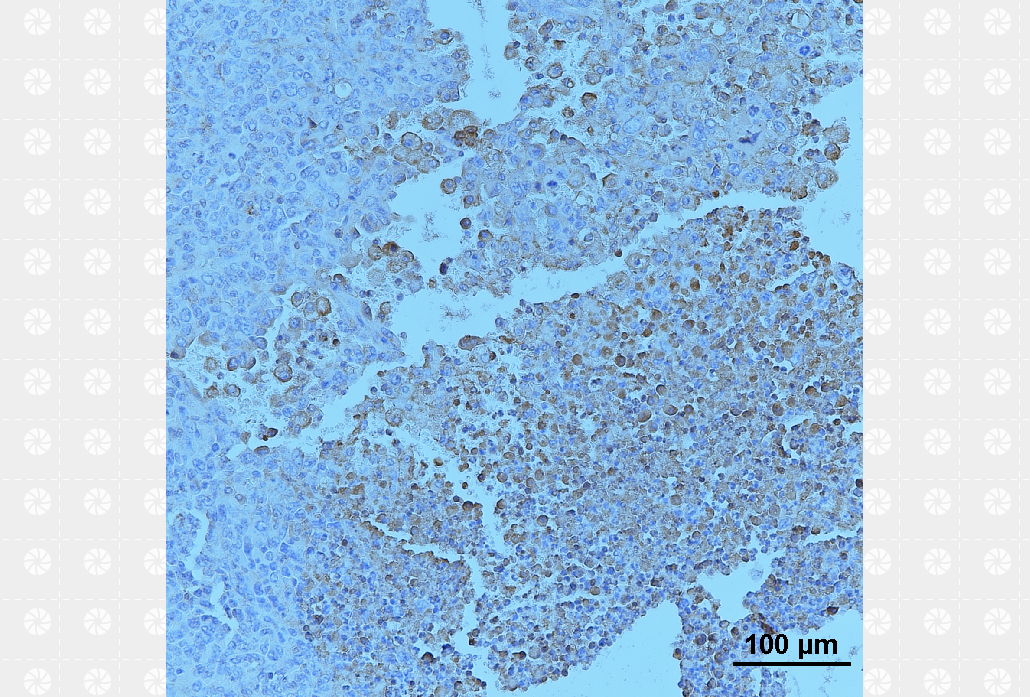

Supplement: Supplementary file 4 [file Data_Sheet_2.ZIP › figure 6 original images/shTGFβ1.CD8.tif]

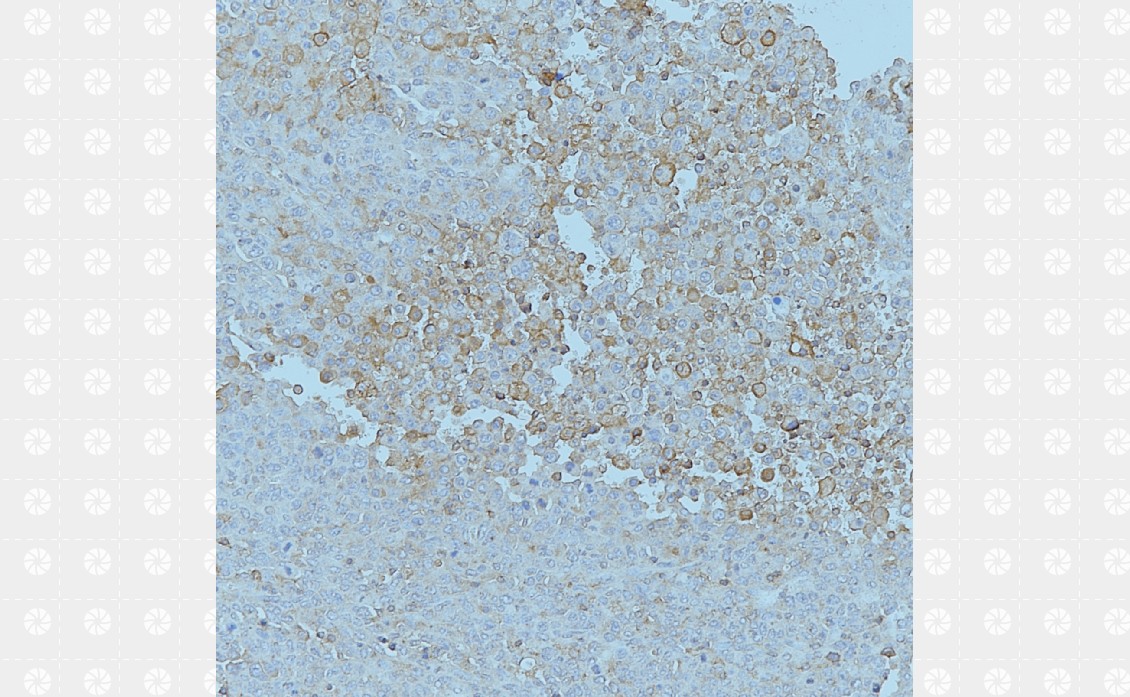

Supplement: Supplementary file 4 [file Data_Sheet_2.ZIP › figure 6 original images/shTGFβ1.CD86.jpg]

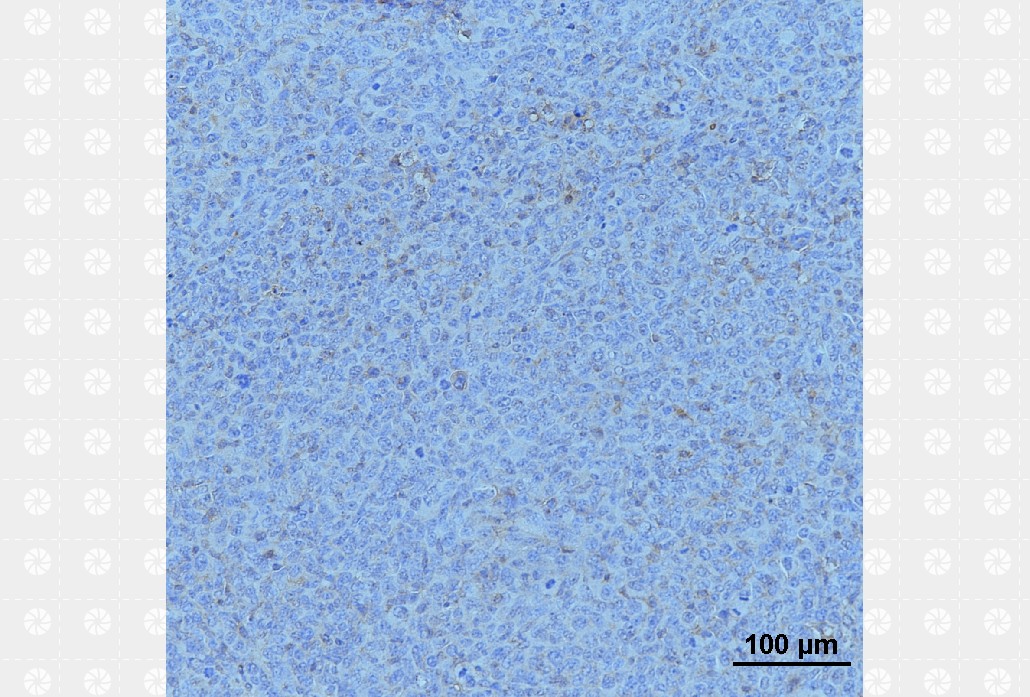

Supplement: Supplementary file 4 [file Data_Sheet_2.ZIP › figure 6 original images/shTGFβ1.FOXP3.jpg]

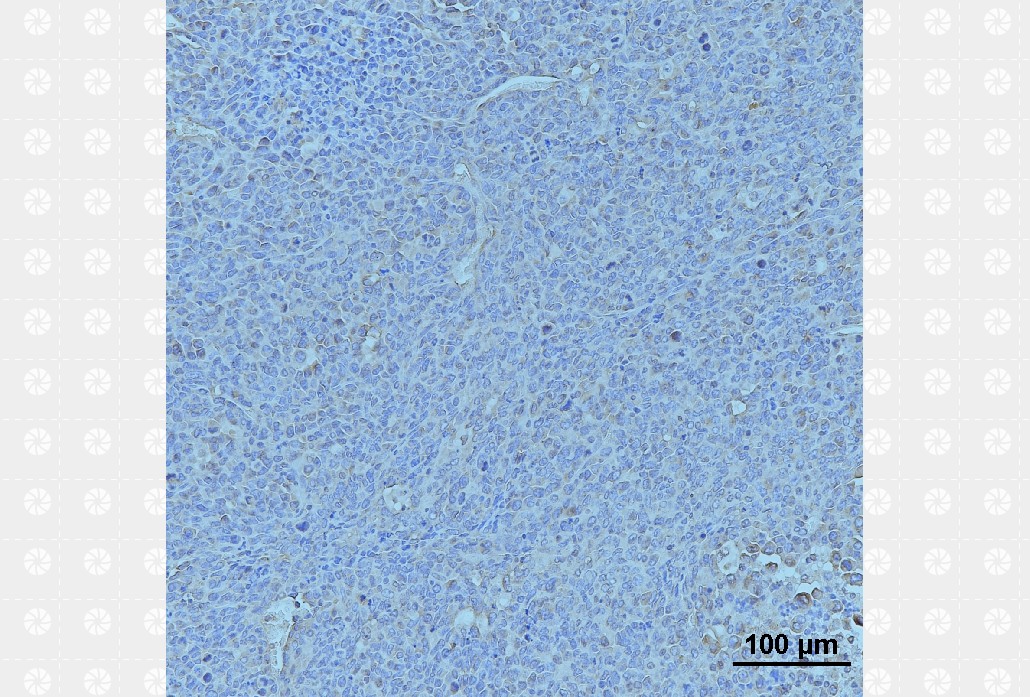

Supplement: Supplementary file 4 [file Data_Sheet_2.ZIP › figure 6 original images/shTGFβ1.PDL1.jpg]

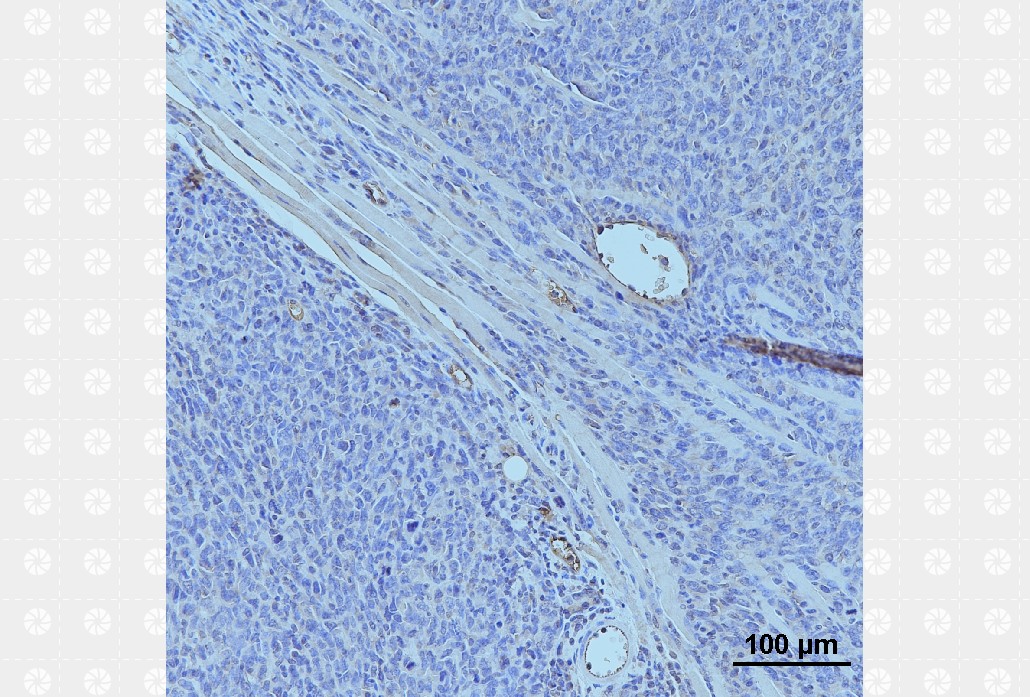

Supplement: Supplementary file 4 [file Data_Sheet_2.ZIP › figure 6 original images/shTGFβ1.TGFβ1.jpg]

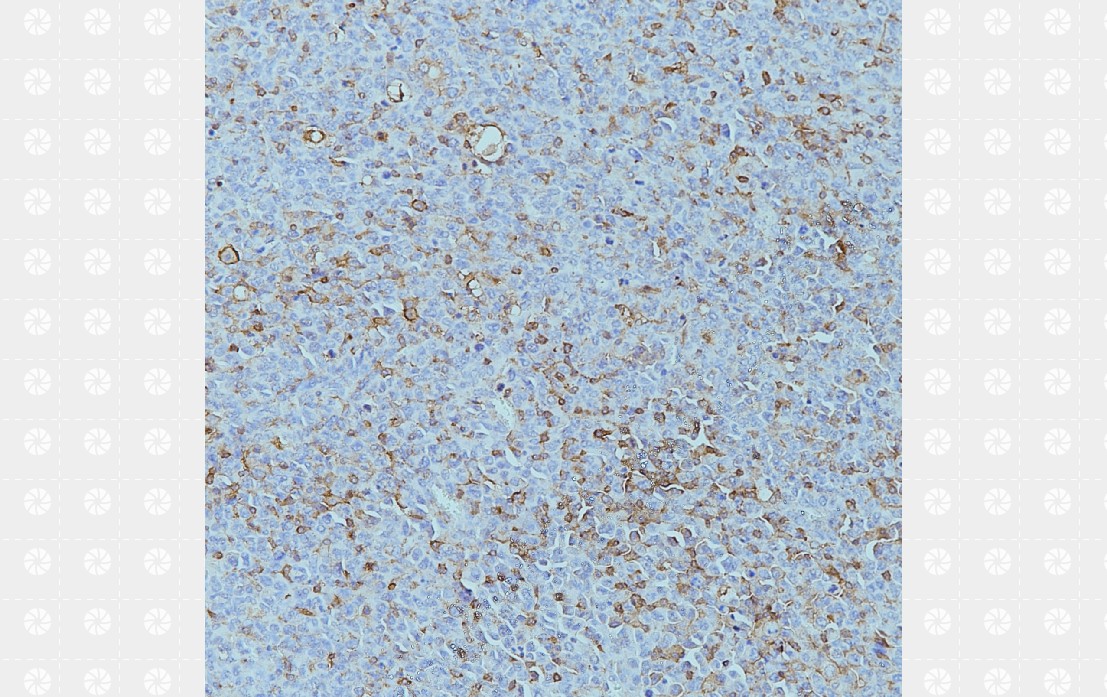

Supplement: Supplementary file 4 [file Data_Sheet_2.ZIP › figure 6 original images/TGFβ1ox.CD11b.jpg]

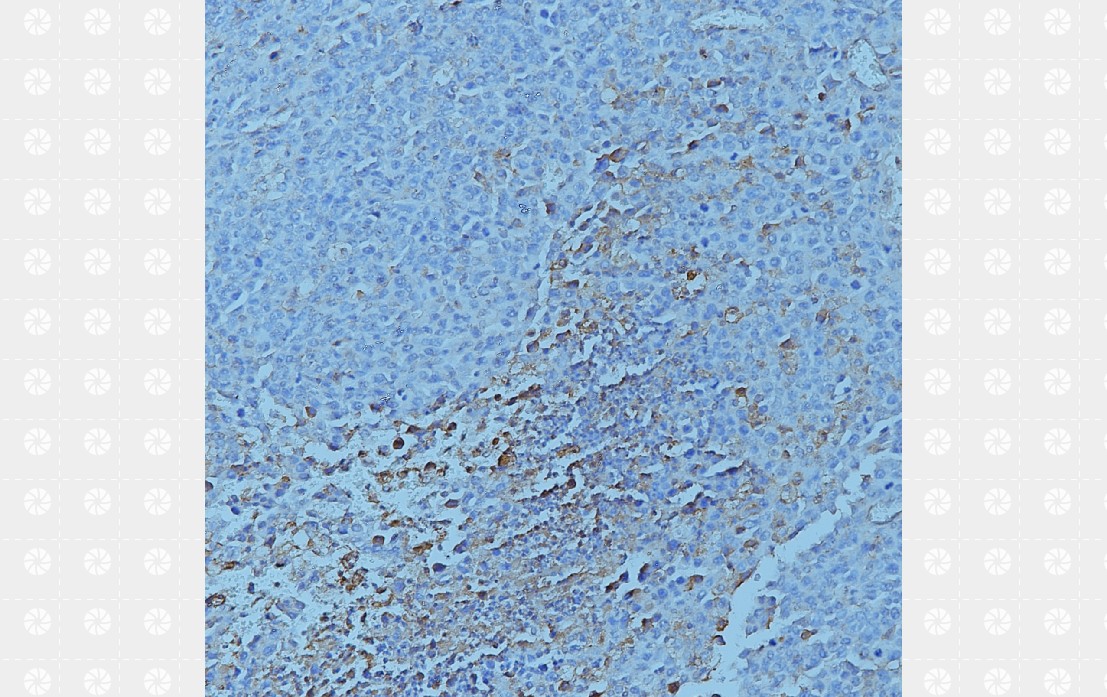

Supplement: Supplementary file 4 [file Data_Sheet_2.ZIP › figure 6 original images/TGFβ1ox.CD206.jpg]

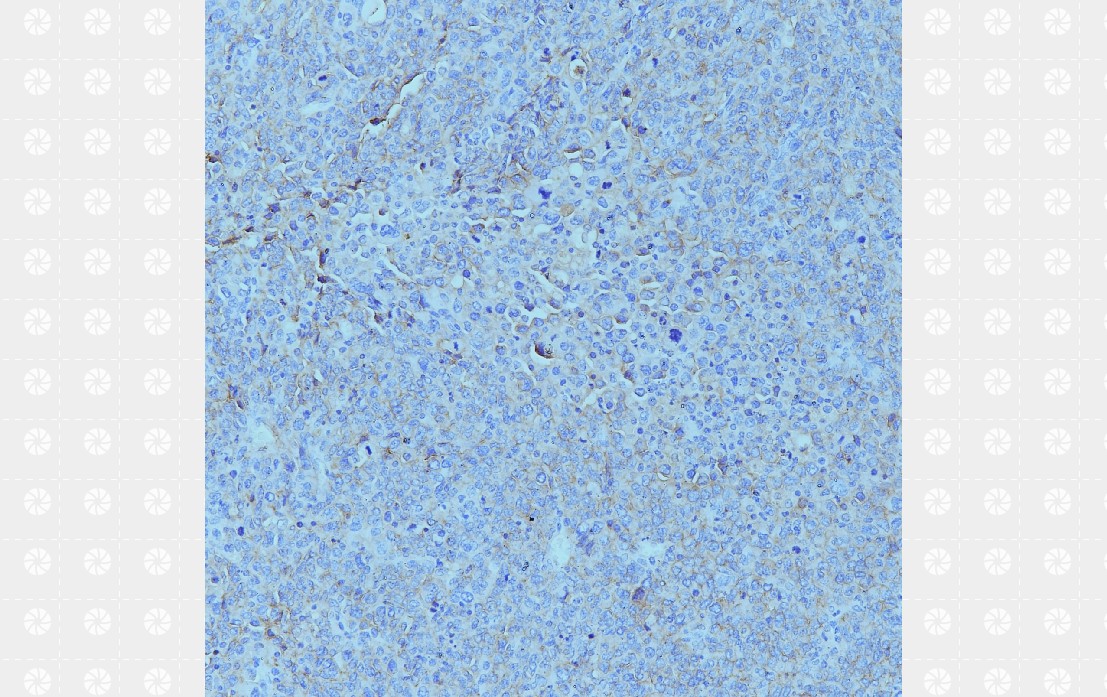

Supplement: Supplementary file 4 [file Data_Sheet_2.ZIP › figure 6 original images/TGFβ1ox.CD56.jpg]

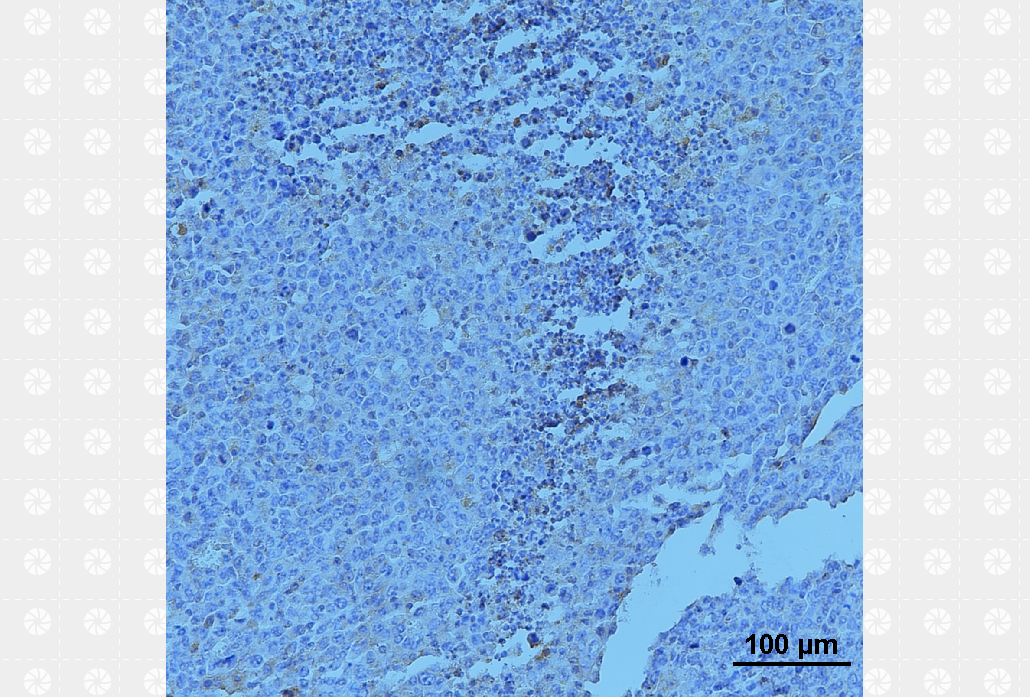

Supplement: Supplementary file 4 [file Data_Sheet_2.ZIP › figure 6 original images/TGFβ1ox.CD8.tif]

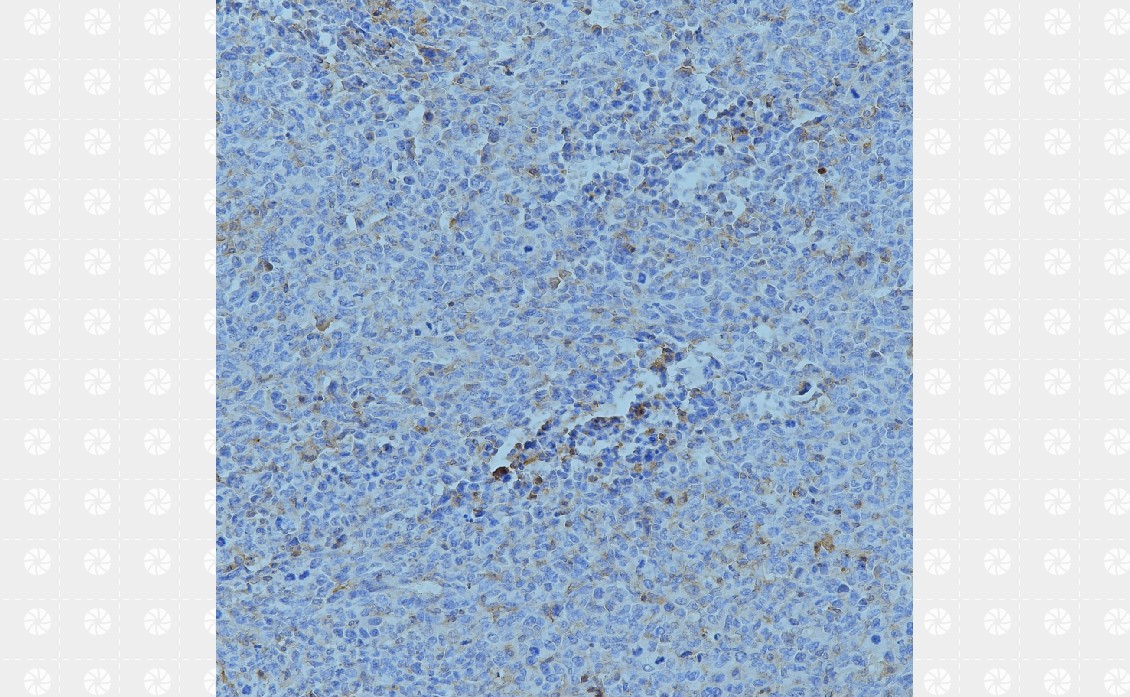

Supplement: Supplementary file 4 [file Data_Sheet_2.ZIP › figure 6 original images/TGFβ1ox.CD86.jpg]

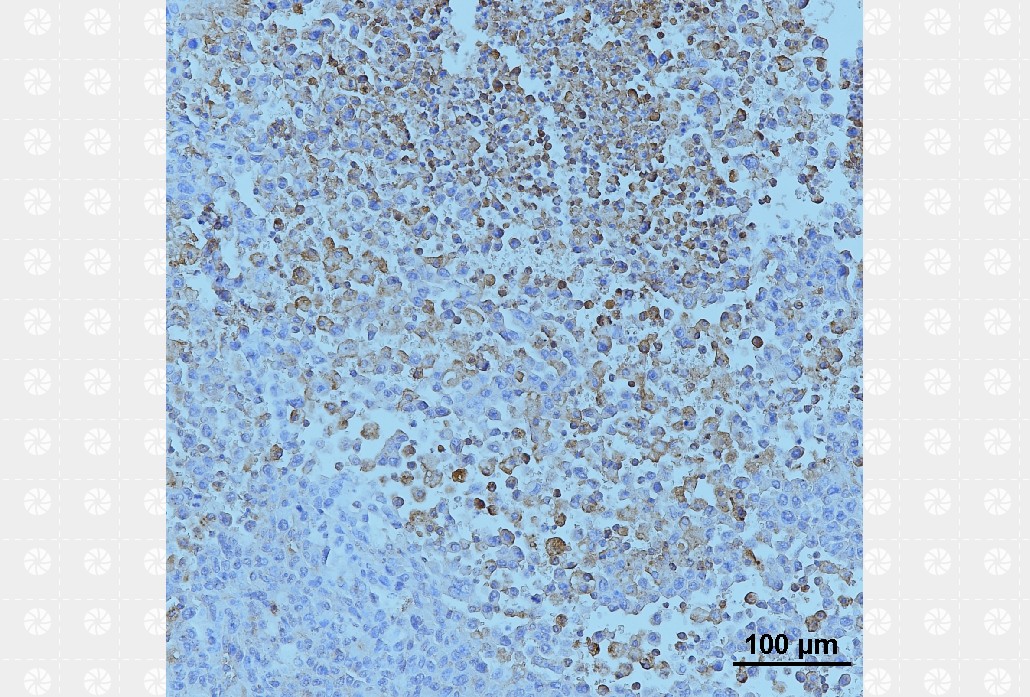

Supplement: Supplementary file 4 [file Data_Sheet_2.ZIP › figure 6 original images/TGFβ1ox.FOXP3.jpg]

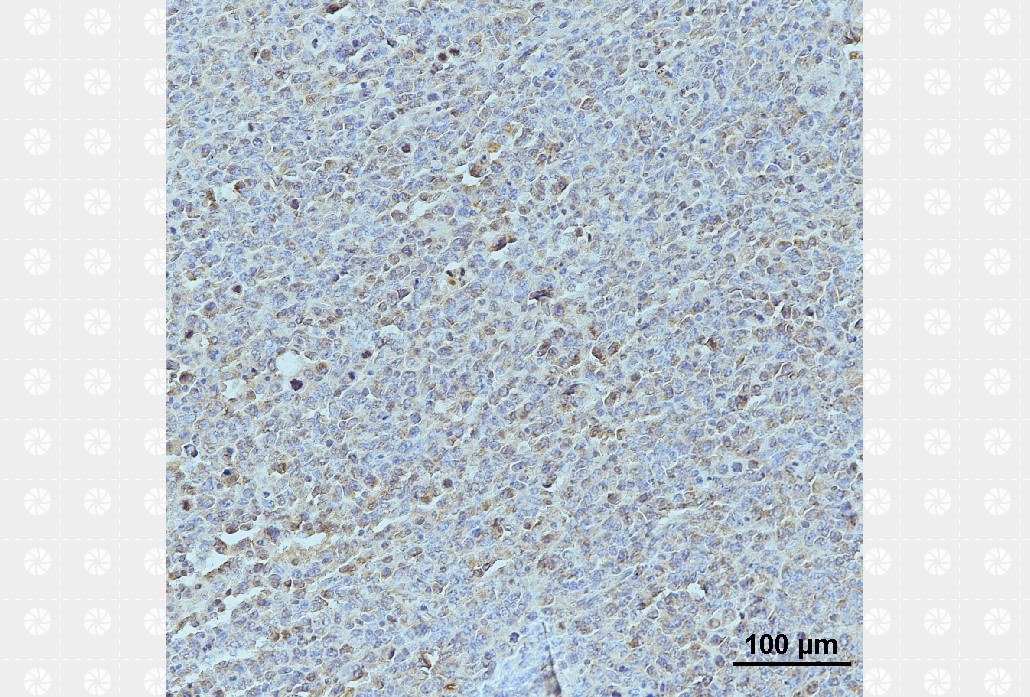

Supplement: Supplementary file 4 [file Data_Sheet_2.ZIP › figure 6 original images/TGFβ1ox.PDL1.jpg]

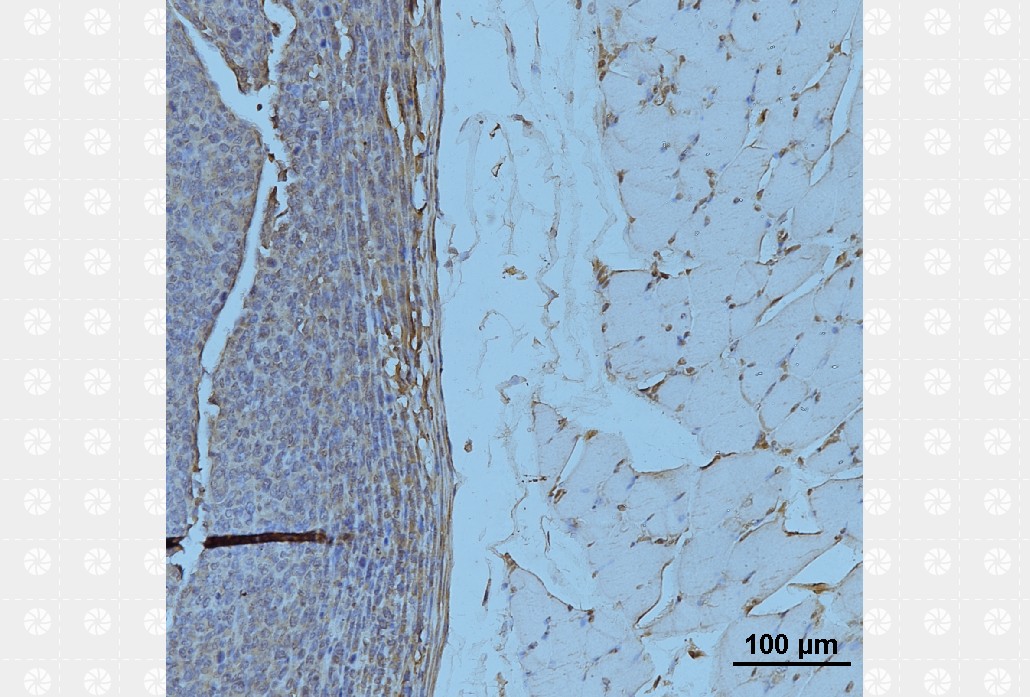

Supplement: Supplementary file 4 [file Data_Sheet_2.ZIP › figure 6 original images/TGFβ1ox.TGFβ1.jpg]
